# A systematic review of the early dialogue frameworks used within health technology assessment and their actual adoption from HTA agencies

**DOI:** 10.3389/fpubh.2022.942230

**Published:** 2022-10-06

**Authors:** Nora Ibargoyen-Roteta, Lorea Galnares-Cordero, Gaizka Benguria-Arrate, Kelly Rocío Chacón-Acevedo, María Paula Gutiérrez-Sepulveda, Eduardo Low-Padilla, Ilich Herbert De La Hoz-Siegler, Claudia Isabel Guevara-Pérez, Ángel del Pozo-Pérez, Marta Suárez, Hans Peter Dauben, Maximilian Otte, Iñaki Gutiérrez-Ibarluzea

**Affiliations:** ^1^Basque Office for HTA (Osteba)/Basque Foundation for Health Innovation and Research (BIOEF), Barakaldo, Spain; ^2^Translational Research Group, Global Institute for Clinical Excellence, Keralty SAS, Bogotá, Colombia; ^3^Biokeralty Research Institute AIE, Miñano, Spain; ^4^dkHealth Partners, Cologne, Germany; ^5^International HealthTechScan (i-HTS), Cologne, Germany; ^6^Basque Foundation for Health Innovation and Research (BIOEF), Barakaldo, Spain

**Keywords:** early dialogue, scientific advice, early advice, systematic review, technology assessment, biomedical

## Abstract

**Introduction:**

Early advice in the process of developing health technologies allows manufacturers to plan their production and transfer to health care systems more accurately. This review aims to describe frameworks used within HTA and their current use by HTA Agencies.

**Material and methods:**

We carried out a systematic literature review in Pubmed, Embase, Scopus, and WoS, including all references published in Spanish and English. This was last updated in March 2022. We extracted all available information regarding the organizations involved, services offered, types of technology, collaborators involved, fees, output and impact. Websites of several HTA organizations and Google were also searched in order to update and complete the information obtained from this generic search.

**Results:**

Five-hundred and forty one articles were identified and screened, of which 26 met the eligibility criteria and were selected. Seven of them were non-systematic reviews that described two or more HTA organizations. Ten studies were focused on the advice offered by individual organizations, and eight described the EMA and EUnetHTA parallel or joint advice. We found variations in the technology assessed, services offered, stage of development and costs for advisory services.

**Conclusions:**

Early and scientific advice would help manufacturers focus their product development on what is needed for the management of specific diseases. Most of the examples or services found refer to drugs as well as to some medical devices and diagnostics. A common definition of the type of advice that could be offered for different health technologies by HTA bodies to ascertain health care systems and manufacturers' needs, in addition to the timeline in which that advice needs to be given, would help HTA bodies provide the right support at the right time.

**Systematic review registration:**

https://www.crd.york.ac.uk/prospero/display_record.php?ID=CRD42020219401, PROSPERO CRD42020219401.

## Introduction

Innovators must ensure that the health technologies they are developing comply with not only regulatory purposes, but also add value to health care systems in terms of additional diagnostic/therapeutic benefit compared to those already available. A study of competing services and technological costs along with regulatory and reimbursement requirements will then be necessary to aid their introduction into health care systems (1). Nevertheless, many of these initiatives are not likely to succeed due to the lack of added value or because all the steps from regulation to market access have not been well planned, leading the technology down a blind alley (1, 2).

Indeed, existing systems have been centered around regulatory requirements which have been increasingly focused on swift patient access, but ignoring the perspective of HTA/payers. On many occasions, this situation has led payers to the very uncomfortable position of either having to reject a potentially effective drug or other health technology or accept a high degree of uncertainty.

Early Dialogue (ED)/Scientific Advice (SA)/Early Advice (EA) has been considered as a well-defined, systematically applied, structured process to provide evidence required for decision making in a certain system for health-related needs. This is based on specific indications and an appropriate research plan and forecast for the development of pharmaceuticals, medical devices and diagnosis.

In recent years, Health Technology Assessment (HTA) organizations have tried to encourage the participation of other stakeholders in their outputs and initiatives. Many HTA bodies have participated in ED or SA with manufacturers, alone or in collaboration with regulatory agencies (3). These processes have been mainly defined as the “*SA given before the start of pivotal clinical trials (drugs), to improve the quality and appropriateness of the data produced by the developers because of a future assessment for reimbursement*” (3).

Manufacturers have welcomed these initiatives because they perceived HTA as an obstacle or hurdle in order to access health care systems (3). Payers usually use HTA assessments to support reimbursement decisions even though the evidence that they need to make those decisions is not always available. Moreover, the information they need may also depend on the context. Therefore, companies have to plan how to obtain the evidence that addresses HTA and payers' needs effectively so as to avoid an unfavorable reimbursement decision in the future, a delay in the introduction of the new technology or even complete withdrawal from the market (4). All HTA bodies use similar approaches for evidence search and its evaluation, but different social values, context related aspects (standards of care, priorities…) or economic issues could lead to divergences in coverage decisions in different countries. ED/SA can help manufacturers to meet the evidence requirements better for their reimbursement, but it does not ensure universal access to all health care systems (5).

In 2009, some European HTA bodies introduced the possibility for manufacturers to ask for advice about their evidence generation plans that would satisfy the payers' and HTA evidence requirements better. Some reviews have been published describing and comparing some of the initiatives put in place by HTA organizations. Unfortunately, not all organizations offering those services were included in those reviews. Moreover, newly established initiatives and their processes have not been included.

This review aims to identify, through a systematic review, all the ED/SA frameworks used within HTA, their characteristics and current level of adoption by HTA Agencies.

## Materials and methods

### Systematic literature search of ED/EA/SA frameworks

To start with, a systematic review was conducted following the “*preferred reporting items for systematic reviews and meta-analyses*” (PRISMA) statement (6). The protocol was submitted to the International Prospective Register of Systematic Reviews (PROSPERO) and it was registered as CRD42020219401.

The research question was the following: Which ED/SA/EA processes are being used by HTA bodies and what has been their overall aim in terms of the number of advice processes developed, types of technology addressed, final assessment, appraisal or reimbursement decision results obtained, as well as the level of satisfaction with the service provided from manufacturers, HTA researchers and decision makers?.

#### Eligibility criteria

All the articles, reports and documents that employed an ED/SA framework used in HTA or health services' research, were considered eligible for inclusion. On the other hand, articles, reports and documents describing ED/SA/EA processes produced only by Regulatory Agencies were excluded. All the articles that measured the impact of the ED/SA/EA frameworks in terms of product improvement, acceptance and added value, as well as the developers' satisfaction with the ED/SA/EA process were also included. Those aspects were defined to assess the value of the ED/SA/EA services offered and also to help identify the issues that should be overlooked to improve the process where necessary.

#### Search strategy

The search strategy was performed in PubMed, Scopus, Web of Science and Embase databases in order to retrieve potentially eligible articles. A generic search was carried out in October 2020, with the last search update in March 2022 for studies published in Spanish or English. There were no restrictions regarding the type of study design although it excluded notes, errata, and/or letters.

The search query used in Pubmed was as follows:

(“health technology“[Title/Abstract] OR ”technology assessment“[Title/Abstract] OR ”policy making“[Title/Abstract] OR ”health policy“[Title/Abstract] OR ”decision making“[Title/Abstract] OR “regulatory[Title/Abstract] OR regulation”[Title/Abstract]) AND (“scientific advice”[Title/Abstract] OR “early dialogue”[Title/Abstract] OR “early advice”[Title/Abstract])

In the case of the other databases, the PubMed query was adapted according to each database's specific search criteria (see Supplemental material 1).

In addition, to update and complete the information obtained in the literature from the generic search, searches were made on the websites of those HTA organizations that were mentioned in the selected articles (not only reviews, but also conference abstracts or individual organization descriptive or methodological reports) as well as on Google, in order to find relevant or updated information not provided in the obtained articles.

#### Study selection and data extraction

All the identified articles were uploaded to the Reference Manager software, and duplicates and unnecessary study design references were removed.

Six independent researchers (LG-C, NI-R, GB-A, KC-A, MG-S, and ID) started the screening process of the articles based on titles and abstracts. Subsequently, selected articles with full-text availability and conference abstracts were carefully read by pairs (LG-C, MG-S, NI-R, ID, GB-A, and KC-A). It was then decided to include articles published from 2011 onwards, despite the fact that some organizations started ED/SA/EA processes in 2009. A 10-year timeframe was considered to be long enough to identify all relevant initiatives and their current characteristics. The articles and conference abstracts which met the eligibility criteria were selected for inclusion. A PRISMA flow chart was used to record all the steps followed. Any disagreement was resolved by consensus and discrepancies were discussed with a third researcher (IG-I).

Two researchers (NI-R and LG-C) performed the data extraction from all the selected articles. The following data were gathered in a specific extraction sheet designed by IG-I for that purpose:

- Reference (author and year)
- Objective
- Organization/s mentioned and their characteristics
- Type of technology (drugs, medical devices, diagnostics…)
- Material and methods (used in the article)
- Results (type of services offered; impact assessment of scientific advice offered etc.)
- Terms used to describe the process
- Other considerations.

In order to organize the information obtained about the ED/SA/EA process used by each HTA body (from each organization's website as well as from the articles included), a table was designed with the following characteristics: organization involved, articles in which the organization is mentioned, services offered, types of technology, description of advice services (individual or parallel advice with regulators and other bodies, steps and timelines), stakeholders involved (who and how they participated), fees - if any, output and impact (if articles identified them). The information for each organization was obtained by two groups of researchers (LG-C, NI-R, GB-A, KC-A, MG-S, and ID) and any disagreement was resolved by reaching a consensus.

Due to the descriptive nature of this systematic review, no quality assessment or meta-analysis of data was carried out for the selected studies.

## Results

### Search results

**Figure 1:**
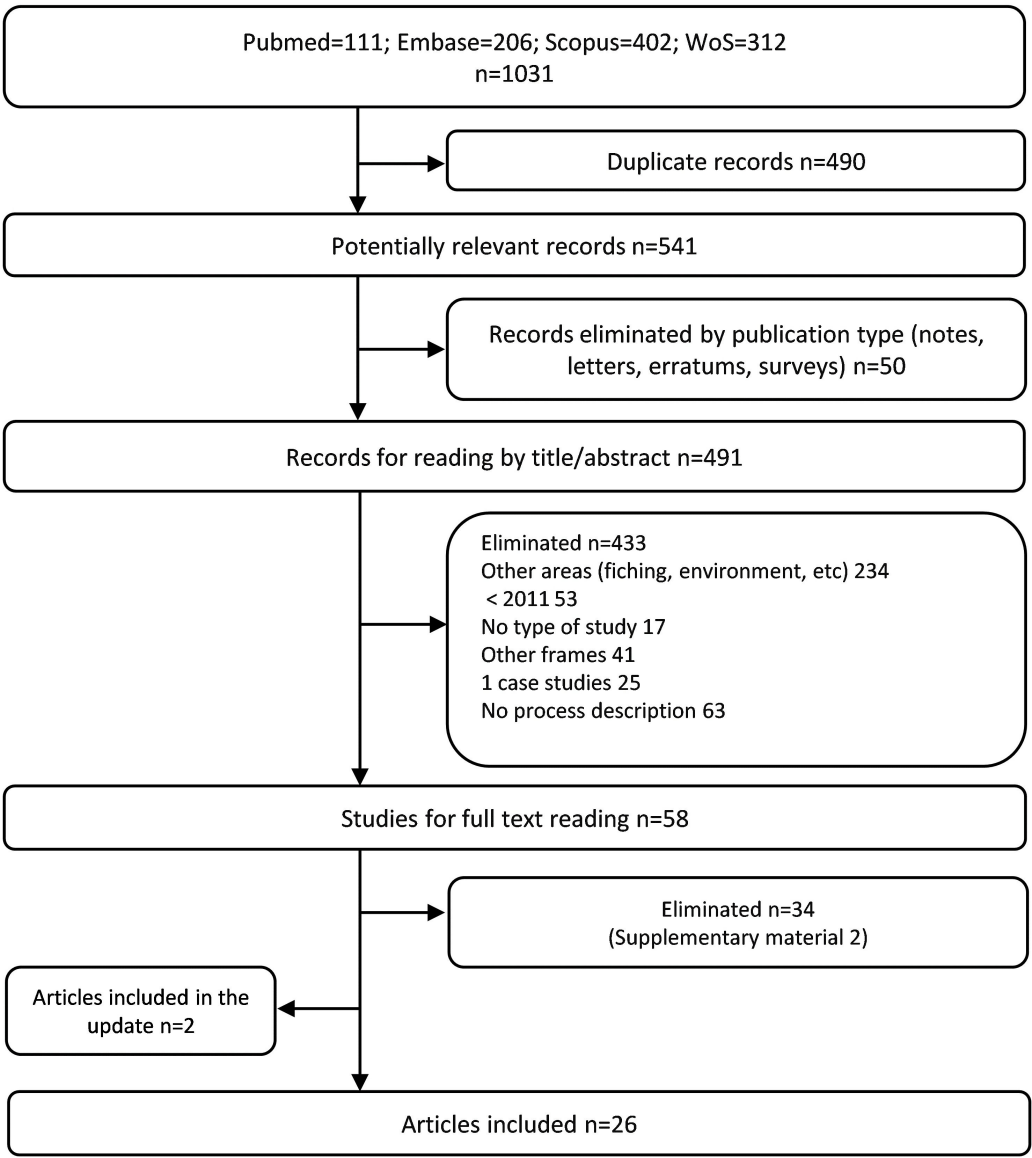
Preferred reporting item for systematic reviews and meta analyses (PRISMA) flow diagram of the study selection process.

The initial search on PubMed, Embase, Scopus, and WoS identified a total number of 1,031 articles. After removing the duplicates and studies which did not match the publication type, 541 articles were finally screened by title and abstract. Fifty eight full-text articles were carefully read, of which 24 met our inclusion criteria (4, 7–29). Another 34 were excluded due to the type of information they provided, such as not describing the framework or reporting single technology advice experiences (see Supplementary material 2). Two more articles were added from the search update (30, 31). Finally, a total of 26 articles (4, 7–31) were included in the systematic review. All the screening and selection process is detailed in Figure 1.

### Characteristics of the selected studies

Of these 26 articles, seven were narrative or non-exhaustive systematic reviews describing the approaches for ED/SA followed by different HTA organizations (4, 10, 15, 17, 19, 21, 24) (see Supplementary material 3). From those reviews, four (14, 18, 20, 23) were conference abstracts, so not much information could be obtained from them. Nevertheless, those reviews were used to identify the HTA organizations that offered formal ED/SA procedures.

On the other hand, 10 studies focused on the SA offered by a single organization (7, 12–14, 16, 22, 23, 26–28). Most of them are related to the characteristics of the SA given by the National Institute for Clinical Excellence (NICE) at different periods (6, 26, 27) or for a specific type of health technology (16). References to the *Gemeinsame Bundesausschuss* or Federal Joint Committee G-BA (23, 28) and the *Haute Autorité de santé* (HAS) (12), among others, have also been identified (see Supplementary material 3).

The websites of those HTA bodies identified by the search strategy as offering ED/SA services were consulted to check for updates and for more complete information which was not published in the articles.

Nine associated articles were identified in the European Network for Health Technology Assessment (EUnetHTA) – (8, 9, 11, 18, 20, 25, 29–31), of which most related to the parallel advice given by the European Medicines Agency (EMA) and EUnetHTA (see Supplementary material 3). EUnetHTA's website was also consulted to verify that there were no current changes in the services offered and defined in the articles.

### ED/SA/EA processes

The HTA ED/SA/EA processes are relatively new services and are frequently updated by the organizations that offer them. We identified the services that were still active at the time this study was developed and updated the information which was last published on each organization's website.

The HTA bodies working on ED/SA/EA approaches were mostly based in Europe (NICE, HAS, G-BA…), followed by Canada [Canadian Agency for Drugs & Technologies in Health (CADTH)]. Most of these organizations not only have a national public service for ED/SA, but also participate in joint or parallel advice services in collaboration with regulatory agencies, at a national or international level, or with other HTA organizations (17). For this reason, the description of the services has been structured per organization. Tables 1, 2 show the organization, the type of services that they offer and the type of technology for which the services are offered, but more complementary information is given in Supplementary material 4.

**Table 1:** Scientific advice/early dialogue services by individual HTA bodies.

**HTA body (references)**	**ED/SA/EA**	**Technology**	**Duration (weeks)**	**Regulatory/other HTA bodies involved**	**Involved stakeholders**	**Fees**
NICE (4, 7, 14, 16, 17, 19, 21, 24, 26, 27)	Standard SA Express SA	Pharmaceuticals Medtech (META tool)	12–18	Individual or in parallel with CADTH/ regulatory EUnetHTA	Depending on the needs: • a clinician • a health economist • an HTA expert • a patient expert or carer regulators also if required.	£29,000 to 91,051
HTW (Wales) (13)	SA (META tool)	Non-medicine technologies	6–8	Individual	Not reported.	NR
CADTH (14, 15)	SA Early PSA	Pharmaceuticals	18	Individual or in parallel with NICE/Health Canada	Not reported.	65,000–100,000 CAN $
G-BA (4, 17, 19, 21, 23, 24, 28)	Early/late SA	Pharmaceuticals	8	With national approval authorities EUnetHTA	G-BA staff	2,000–10,000 €
HAS (12, 19, 21, 24)	ED Standard or accelerated	Innovative medicinal products/ medical devices	11–16	Individual EUnetHTA	HAS staff. Experts and patients at request.	No fees.
ZIN (4)	SA (written or oral advice)	Medicinal products	6–12	In parallel with MEB EUnetHTA	MEB staff. Clinicians and ZIN staff could participate	No additional fee to MEB
TLV (4, 17, 21)	SA	Pharmaceuticals	8	In parallel with MPA EUnetHTA	MPA staff. Company can ask for more experts, including TLV staff.	No additional fee to MPA
AIFA (10, 19, 21)	SA *Innovative meetings*	Pharmaceuticals	12	Individual EUnetHTA	AIFA staff.	10,000–40,000 €

*HTA, Health Technology Assessment; ED, Early Dialogue; SA, Scientific Advice; EA, Early Advice; NICE, National Institute for Clinical Excellence; HTW, Health Technology Wales; CADTH, Canada's Drug and Health Technology Agency; G-BA, Federal Joint Committee; HAS, Haute Autorité du Santé; ZIN, the National Health Care Institute; TLV, The Dental and Pharmaceutical Benefits Agency; AIFA, Agenzia Italiana del Farmaco; EUnetHTA, European Network for Health Technology Assessment; MEB, Medicine Evaluation Board Agency; MPA, Swedish Medical Products Agency, NR, Not Reported; CAN, Canadian.

**Table 2:** Early dialogue/joint advice services by EUnetHTA and EMA.

**HTA body (references)**	**ED/SA/EA**	**Technology**	**Duration (weeks)**	**Regulatory/ other HTA bodies involved**	**Involved stakeholders**	**Fees**
EUneHTA-EMA (4, 8, 11, 17, 18, 20, 25, 29–31)	ED	Drugs and medical devices	Eight for written format 11 for F2F format	Multi-ED Parallel ED with EMA	EMA HTA bodies Patients/clinicians	EMA' fees and some HTA bodies can charge fees

*HTA, Health Technology Assessment; ED, Early Dialogue; SA, Scientific Advice; EUnetHTA, European Network for Health Technology Assessment; EMA, European Medicines Agency; F2F, face to face.

#### ED/SA/EA services offered by each HTA body

##### National institute for health and care excellence (NICE), UK

NICE was mentioned in 12 of the studies included in this systematic review (4, 7, 10, 14–17, 19, 21, 24, 26, 27). All the reviews identified and compared NICE with other HTA organizations offering ED/SA/EA (4, 10, 15, 17, 19, 21, 24). Four other references described the SA offered by NICE since 2009 (when the service started) (7, 16, 26, 27), and one of these described and compared the process with the one offered by EUneHTA (14).

Updates on the SA services offered by NICE were consulted on its website (32), confirming that currently, NICE offers a different kind of SA to manufacturers, depending on the timeline, the type of health technology and its stage of development.

For NICE, the optimum time to seek advice on the clinical development and evidence generation plan of pharmaceuticals is during the design period and before the initiation of the main studies of safety registration and efficacy. However, this could vary depending on the technology. Nevertheless, companies should be aware of the need to have sufficient time to act on advice given at any time. In the case of medical devices and diagnostics, there is generally more variation, and NICE encourages manufacturers to get in touch to discuss the ideal time to seek advice (32).

For pharmaceuticals, depending on the timeline, two main types of advice services are available: standard and express. Both types of advice start at week 1, when NICE confirms the project fee. For standard SA, in week 10, a three-h face to face meeting takes place with NICE and a panel of experts, and NICE submits the subsequent advice report in week 18.

For express advice, the meeting takes place in week 6 and the report is sent in week 12 (14).

For Medtech products, NICE offers a hybrid service. Manufacturers can ask for a META (Medtech Early Technical Assessment) tool consultation (33), based on a platform developed by NICE, which involves a face to face discussion between the developer and the adviser to identify any gaps in the development and evidence generation plans of the product. For more advanced (higher level in the Technology Readiness level TRL score) Medtech products, META Tool consultation could be combined with the more traditional SA service, although this is not specified. In this case, both standard and express advice are available.

NICE fees for standard advice range from £38,024 to £91,051; for express advice from £49,431 to £82,632 and concurrent advice from £31,221 to £84,397. There are fixed fees for MedTech Advice (£15,000) and META Tool consultation (£3,500).

It should also be mentioned that small and medium-sized enterprises can discuss more affordable options (32).

Maignen et al. (7) analyzed all the SA for investigational medicinal products in which NICE had participated from 2009 to 2015 and observed that questions related to the clinical development and, specifically, to the main pivotal efficacy studies, were the most frequently addressed questions. When considering the questions raised in parallel advice procedures with EMA and HTA bodies, procedures generally focused on clinical efficacy issues, whereas cost-effectiveness issues tended to prevail in NICE-only procedures. Their analysis showed that the most frequently addressed issues by the SA were the selected comparator, the generalisability of the clinical trial evidence to the National Health Service (NHS) practice, and the impact that outcomes would have on patients' survival and quality of life. Less variation was found concerning the choice of clinical endpoints, the definition of the population, the importance of technology in the treatment procedure, and the design of the study (7). No further studies describing the impact of the SA offered by NICE have been identified in the research.

#### Haute autorité de santé (HAS), France

Three articles mentioned HAS as a single HTA organization offering ED/SA (19, 21, 24). The French Social Security Code (CSS) states that one of HAS's missions is to organize EDs with companies that develop innovative medicinal products or other technologies with a new mechanism of action. These are aimed at insufficiently covered medical needs or if the request is submitted before the start of pivotal clinical trials (34). The final objective is to give recommendations to companies about the latest phase of development of the technology (pivotal study/studies), answer medical and medico-economic questions and support the generation of good quality evidence from the HTA's perspective. The EDs offered by HAS are optional, confidential and not binding (for both HAS and the companies).

For innovative medicines, there are two different ED procedures: (a) a standard procedure (with a face-to-face meeting) or (b) an accelerated procedure (without a face-to-face meeting). In the case of medical devices in clinical development, HAS provides recommendations and answers to better anticipate the type of clinical data they will need to provide to meet the requirements of HTA (35).

Confidentiality is guaranteed during the process. On the other hand, there must be no conflict of interest with the technology being assessed. The applicant also agrees to the same rules of confidentially in HAS's final written recommendations.

Experts and HAS staff who have participated in the EDs are not allowed to participate in any future assessments and appraisals of the technology and vice versa.

By the end of 2018, HAS had participated in a total of 84 EDs (53 of them in collaboration with EMA and/or other European HTA bodies), and mainly focused on medicinal products' phase III trials (12). Following the ED, the clinical study for which the company asked for advice had not yet been implemented in 25 cases. In the 50 cases in which the clinical trial was effectively launched, the authors pointed out that the results were negative (unfavorable to the product) in 10 cases, positive (proving the expected benefits of the product) in 11 and ongoing in 29 cases. Clinical development was officially withdrawn or suspended before the initiation of the trial in nine cases. Overall, only eight medicinal products were appraised by HAS, all of them obtaining a clinical added value score. The authors stressed that the success rate of the products that were part of an ED procedure was higher than that found in the published literature.

#### AIFA, Italy

Three articles mentioned AIFA as an HTA organization offering ED/SA services (10, 19, 21). On the agency's website, two kinds of services are described, even though the temporary suspension of these services was announced on 21 December 2021.

In Italy, a process for both SA and HTA advice was introduced in 2011 (10). AIFA offers “*a fee-formal national early HTA advice (separate from regulatory advice and with the official description of the process and application form)”* (21), with the production of a final written report.

It was indicated that “*the SA-HTA advice is generally asked for a single product at early stages of development, but it may be requested also for broader therapeutic classe*s”. In general, they refer to products in Phase II and Phase III. The issues that can be addressed in the advice include the definition of the most suitable comparator(s), the outcomes to be measured, the acceptability of indirect comparison and the target population.

The fee can range from €10,000 to €40,000, depending on the questions asked (4). The output of the SA is a final report sent 90 days after signing the contract. The current uptake of this service by manufacturers in Italy was not publicly available at the time of writing (10). No article assessing the impact has so far been identified.

Like many other organizations, AIFA has participated in Parallel Scientific Advice (PSA) through EUnetHTA, and is the third most involved agency from 2010 to 2015 (8).

More recently, AIFA has been offering so-called “innovative meetings”, which are defined as “*informal meetings during which it is possible to present an innovative product, technology or methodology to receive feedback or guidance on the evolution of the development program*” (36), but no more information about that service has been found.

#### Federal joint committee (Gemeinsamer Bundesausschuss, G-BA) and institute for quality and efficiency in health care (Institut für Qualität und Wirtschaftlichkeit im Gesundheitswesen, IQWiG), Germany

In Germany, the HTA SA for pharmaceutical manufacturers is organized by the Federal Joint Committee (FJC) or G-BA, within the early benefit assessment process (EBA) for pharmaceuticals. The FJC commissions the Institute for Quality and Efficiency in Health Care (IQWiG), an independent HTA-supporting scientific institute which prepares evidence reports on pharmaceuticals and non-drug interventions (28).

FJC was mentioned in four of the articles included in the systematic review (4, 19, 21, 24). It advises pharmaceutical manufacturers on request, based on submitted relevant documents.

The whole process includes the FJC's implicated units, the subcommittee for pharmaceuticals, and the specific working group for the EBA, among others.

The Federal Institute for Drugs and Medical Devices (BfArM) as well as the Paul Ehrlich Institute (PEI) (the national approval authorities) can be involved in the SA process. SA can either be “early” or “late”, depending on the possibility of changing the product development plans (if the pivotal trial has not been started).

In relation to the fees, the initiation of the SA requires an advance payment of €5,000. The fee for requests about the appropriate therapy to be compared with is €10,000.

In a study about manufacturers' experience with the SA offered by the FJC (28), a specific questionnaire was sent to the pertinent departments of the members of the German Union of Research-based Pharmaceutical Companies between April 2013 and March 2015. A total of sixty-one questionnaires were completed by 19 manufacturers (25% of the overall SA by the FJC). Fourteen out of 61 were related to early SA (before phase III trials) and 44 (72%) to late SA (ongoing pivotal trials or those already finished). Other stakeholders only participated in 13 cases (21%) in addition to the manufacturer and the FJC. Manufacturers criticized the existence of inconsistencies in the process, the lack of expertise in conducting clinical trials, partially incomplete answers to inquiries and unwillingness to participate in the dialogue. On the other hand, most respondents showed a positive attitude about the unambiguousness, thoroughness, traceability, atmosphere and protocol of the advice. An increasingly positive trend in the perception of the SA by the industry was observed over this time. The authors indicated that “*a more active involvement of additional stakeholders and the incorporation of procedural elements from other healthcare systems could improve the quality of the SA offered by the FJC*” (28).

Plaud et al. (23) only indicated that regarding innovative devices, IQWiG has an ED framework to ensure the fast access of patients to safe and effective medical innovation.

#### Health Technology Wales (HTW), Wales (UK)

In a congress abstract (13), the HTW was identified as an HTA organization that offers SA to developers. The information related to the service offered was obtained through their website. The SA offered by HTW aimed “*to support developers and innovators in Wales to generate evidence and demonstrate the value that meets the needs of care commissioners, care providers, patients, and service users for non-medicine technologies*” (37).

This service could help to (a) identify gaps in evidence (b) support activities to generate evidence (c) save time and resources and (d) reach the market.

HTW uses the NICE META tool for this service and the whole process could take from 6 to 8 weeks.

This is a fee-based service and can be solicited by all-size technology developers of all sizes. HTW has 5 days to decide if the submitted application is within HTWs' scope.

No information about the number of SA offered or their impact on manufacturers' evidence generation plans have been identified.

#### Zorginstituut Nederland (ZINL), the Netherlands

In the Netherlands, ZINL (known before as CVZ) was identified by Cuche et al. (4) as one of the first organizations offering EA, and having also participated in the ED processes undertaken by EUnetHTA (4).

Information about the possibility of asking for both the Medicines Evaluation Board (MEB) (for registration) and the ZINL (for reimbursement) has also been obtained on the MEB website (38). This option could help to design a phase III clinical study that suits both regulatory and reimbursement requirements better. Each organization is only responsible for its own specific advice. For this procedure, the question/s to ZINL can be added in the application form (unless they are related to pharmaco-economic research, which should be submitted to ZINL separately) and all correspondence is directed *via* MEB, who inform whether or not ZINL will participate.

The type of advice provided could be verbal or written (depending on how easy the questions are). Fees before January 2022 could be consulted on their website (39).

#### Dental and pharmaceutical benefits agency TLV, Sweden

In Sweden, TLV was identified as one of the organizations offering HTA advice to companies (4). It offers conjoint regulatory advice with the national regulatory agency. Any company could ask TLV to participate in the SA service offered by MPA (the Swedish Regulatory Agency) (40). The consultation timeframe was 2 months, with a meeting which lasted about 1.5 h. Currently, the SA offered by MPA is about SEK 65,000 (6,239.6€) (40), a fee that does not increase when TLV is included in giving the advice.

#### Canada's drug and health technology agency (CADTH), Canada

In the systematic review, only two articles mentioned CADTH's SA services (14, 15). Boss et al. (15) described all the types of SA services offered by CADTH, announcing two parallel SA programs with NICE (from UK) or Health Canada. Heyes and Millar (14) only mentioned it when describing the possible services offered by NICE. The information has been completed by consulting the CADTH website (41).

The CADTH SA Program (41) offers advice to applicants about their prospective drug development plans, focusing on the development of strategies and the evidence definition requirements that HTA ask for in order to carry out their assessments. The advice is provided by taking the perspective of the Canadian Payer into account. This advice may be requested on clinical, statistical (stratification of a subgroup), or economic topics.

In addition to its standard CADTH-only SA, CADTH also offers PSA with the Canadian regulatory body (Health Canada) or NICE. CADTH SA Program does not offer SA on regulatory aspects, pricing, preclinical studies or the analysis of already existing data. Timelines are similar for both kinds of services. There is a joint SA meeting in week 14 (just with CADTH or also with Health Canada) or week 10 (with NICE). The final report is sent to the applicant 18–20 weeks after submission. The SA fee ranges from $65,000 to $100,000, based on the scale of the project.

#### Pharmaceutical benefits advisory committee (PBAC), Australia

The systematic review identified a single study in Australia about the feasibility of implementing a simultaneous SA process taking both the regulatory and reimbursement perspectives into consideration (22). A pilot study took place in 2009, where advice was needed for two compounds which were possibly to be used in different disease areas. The advice was focused on matters of common interest to the Therapeutics Goods Administration (TGA) and the PBAC (clinical evidence). The developers had to prepare and send the Briefing books (with a proposed clinical development program) 8 weeks before the meeting, and in this pilot study, only verbal advice was provided.

No further current information was identified on their website.

### ED/SA/EA services by EUnetHTA, Europe

In the systematic review, nine references related to EUnetHTA were identified (8, 9, 11, 18, 20, 25, 29–31). The focus of these articles was the description of the EUnetHTA Joint SA in parallel with EMA, except for two references (25, 31) that also included multi-HTA EDs' examples.

The activities of EUnetHTA were organized through the establishment of the EUnetHTA Collaboration 2009 and three consecutive Joint Actions (from 2010 to 2020) (available at: https://www.eunethta.eu/about-eunethta/history-of-eunethta/). The EUnetHTA Joint Action three (2016–2021) developed the final phase to establish a permanent European HTA structure and was succeeded by EUnetHTA 21 (2021–2023), built on the lessons learned (42) and with the aim of supporting a future EU HTA system under the HTA Regulation (43).

ED services to the industry were offered by EUnetHTA in Joint action 3 (42). This service consisted of “*a non-binding SA, before the start of pivotal clinical trials (after feasibility/proof of concept study), to improve the quality and appropriateness of the data produced by the developers given future HTA assessment / re-assessment*” (42). In this case, it was highlighted that there could be HTA-associated fees related to the participation of some HTA bodies.

In February 2021, a call for tender was launched to foster joint HTA work supporting EU cooperation beyond May 2021, thus providing relevant input to the new legal framework on HTA. The contract was awarded to the EUnetHTA 21 Consortium, led by ZINL (the Netherlands) and including other 12 organizations: AEMPS (Spain), AIFA (Italy), AIHTA (Austria), G-BA (Germany), HAS (France), INFARMED (Portugal), IQWIG (Germany), KCE (Belgium), NCPE (Ireland), NIPN (Hungary), NOMA (Norway) and TLV (Sweden). In this case, EMA charges the same fees as for standard SA/Protocol Assistance, and in the case of HTA bodies, it is covered by EUnetHTA 21 budget (43).

#### Multi-HTA ED by EUnetHTA

The aim of multi-HTA ED was to have a collaborative approach among HTA bodies, to exchange views, identify the key issues of the development proposed by the company, discuss their positions and try to reach a consensus on advice as far as possible (25). The first pilots of multi-HTA ED took place in 2012. The “*Shaping European Early Dialogues*” (SEED) consortium (coordinated by HAS) conducted 11 ED (eight in drugs, including four in conjunction with regulatory SA at the EMA and three on medical devices).

Multi-HTA EDs for pharmaceutical products were launched in early 2017. Between June 2017 and May 2021, only six multi-HTA ED for pharmaceuticals were developed by EUnetHTA members (31). Multi-HTA ED was also offered for Medical Devices (EDMD). For that purpose, an EDMD WP (Working Party), composed of AVALIA-T (Spain), HAS (France), NICE (UK), RER (Regione Emilia-Romagna) (Italy) HTA bodies was created. The selection criteria for the medical devices to be included in the process were the following: class IIb/III medical devices (MD), *in vitro* diagnostics, equipment and digital healthcare solutions/connected devices. It was also necessary to address an unmet need, to be top class technology or to have a potential impact on patients, public health or healthcare systems. Besides that, at least three HTA bodies should want to participate in the ED.

#### PSA/Scientific Consultation (SC) from EUnetHTA and EMA

In 2010, the EMA, together with HTA bodies, started a pilot program on PSA. In May 2014, a “Best Practice Guidance for Pilot EMA-HTA Parallel Scientific Advice (PSA) Procedures” was released for public consultation. Formalization came in 2015 (30). Some of the issues included in the guidance document were the following: (1) all medicinal products are eligible (2) the applicant chooses which HTA bodies participate (five HTA bodies maximum) (3) HTA bodies invited to participate were under no obligation to do so (4) a common briefing document was used and (5) advice given was not legally binding. When companies chose not to apply the advice given, they had to justify their position when applying for marketing authorization (6) the process was confidential and EMA was responsible for the administrative work.

An analysis of 43 PSA procedures between 2010 and 1 May 2015 showed that HTA bodies with the highest representation were NICE (90%), followed by G-BA (65%), AIFA (45%), TLV (Sweden) (35%) and HAS (19%) (9).

Full agreements were reached in 61% of the 518 responses provided by the HTA bodies, partial agreements in 23%, and disagreements in 16% (9). Divergence was higher regarding the selection of the comparator (9). In a more recent article, agreement among HTA bodies participating in those processes was around 85% (31).

When analysing the implementation of the recommendations about the choice of comparator, manufacturers implemented both regulators and at least one HTA body's recommendations in 12 out of 21 studies (almost 60%) (8). Studies in which manufacturers followed the regulators' and more than 50% of the HTA bodies' advice were 8/21 (38%), and seven out of 21 followed only the regulatory advice. In all cases manufacturers implemented solely those recommendations made by HTA bodies. For the primary endpoint, manufacturers implemented both the requests of regulators and at least one HTA body in all cases (*n* = 23) and the requirements of both the regulators and of more than 50% of the HTA bodies in 15 out of the 23 cases.

In 2017, the EMA-HTA PSA was substituted by the EMA-HTA parallel consultation (PC) process, this being the key update for the incorporation of the EUnetHTA and ED Working Party (EDWP) (30). Until May 2021, 93 requests for PC were received, from which 32 were finally selected (31).

Participating HTA bodies were fully aligned with the proposed recommendations, with more than 80% of full alignment on all PICO items (31). The authors found that the chance to exchange information between EMA and HTA bodies at various stages before the meeting with the applicant helped to prepare the meeting and added value to the process compared to the previously offered parallel advice procedures. As a result, applicants modified their development plans after receiving the PC list of issues in 12 of the 21 cases analyzed (31).

## Discussion

Given the descriptive nature of the systematic review, no quality assessment has been made for the included studies. The purpose of this review is to produce a global snapshot of all the ED/SA/EA activities of HTA bodies. This global view will serve as a starting point for discussion where the international experiences will be considered in the definition of the services that could be offered by HTA bodies; deciding the best moment to procure advice from the manufacturers; examining the complexity of the processes or how to measure their impact. For example, carefully defining the evidence required for a positive appraisal or a positive reimbursement decision, identifying whether ED recommendations have been implemented or not by the companies involved, or the level of satisfaction of such companies with the ED process.

As mentioned above, the advice given by different HTA bodies to manufacturers varies among organizations, not only in terms of the type of questions that can be addressed, but also the final output resulting from the involvement of external experts (4), the type of technology to be considered, and the stage of development at which the technology should receive advice.

In the beginning, these advice services were mainly offered for innovative products that were in phase II or III studies (mainly drugs), or just before the start of the pivotal trial (medical devices and diagnostics). However, HTA bodies, such as NICE or AIFA, have expanded the types of services offered to companies, to cover the development stage or the type of technology candidate to adapt the type of advice that could be offered (32). In that sense, AIFA offers what they call innovative meetings to the companies, “*during which it is possible to present an innovative product, technology or methodology to receive feedback or guidance on the evolution of the development program*” (36). Therefore, different advice services can be offered depending on the stage of development of the product or the type of technology, and the manufacturers' needs.

Many terms have been used to define these services, and to date, apart from the ED offered by EUnetHTA, no standard terms have been agreed upon. For example, the new HTA Regulation uses the term “Joint scientific consultation” to refer to the exchange of information with health technology developers in their development plans for a given technology, but the definition of that service is not included in Article 2 of the Regulation (44).

Therefore, it would be recommendable for HTA organizations to further define their EA/ED and SA activities and to what extent they align their processes with regulatory advice.

To facilitate discussion, we propose the following terms: HTA advice (any service of ED/SA/EA given to companies by HTA bodies - even other advice could be identified depending on the stage of development of the product); joint advice (the advice given by more than one HTA body, also referred to as joint scientific consultation by the new HTA regulation); parallel advice (the advice given in parallel by HTA bodies and regulators); and regulatory advice (advice given by only regulatory bodies, including so-called scientific advice).

In this systematic review, national HTA bodies who offer an individual HTA advice service have been identified, such as NICE, CADTH, HAS or G-BA. In the case of NICE, differences were identified in the questions posed by manufacturers at a national or European level, for example, in terms of cost-effectiveness. An analysis of the questions dealt with by EUnetHTA in their Joint Action 3, the domain “health economics” is not included as not all members conducted evaluations of health economics (31).

In the recently published results of the advice given in the EUnetHTA Joint Action 3, developed between June 2017 and May 2021, a high percentage of alignment among HTA bodies can be observed, with more than 80 percent fully aligned on all PICO items. Moreover, development plans were modified after receiving the EUnetHTA list of issues in twelve out of twenty-one cases (57%). These modifications included important changes in the design adaptation; the choice of the primary endpoint; the comparator; and the inclusion of additional studies; changes in the target population; the interventions undertaken; and the selection of other outcomes (31). New HTA regulation will try to improve the consensus on the advice given to manufacturers by making it more robust.

With the exception of the publication on G-BA advice (28), no further publications have been identified on the needs and/or satisfaction level of manufacturers with the service/s received. Further analyses on this topic would help to understand different demands (for example, from the initial idea or prototype to post-marketing monitoring) addressing how they could be dealt with by HTA bodies.

In certain countries, manufacturers also have the opportunity to ask for parallel advice at a national level from HTA bodies and regulators. England, Germany, Sweden, and Canada offer this possibility, even if this limits the perspective to one specific context (17). Another example of parallel advice among HTA bodies and the Regulator is that offered at European level and detailed in the recent HTA European Regulation (44). In this case, the advice from HTA bodies may be given in parallel with the SA from the EMA, preserving, in all cases, the separation of their respective remits (from the Coordination Group and the European Medicines Agency). In the case of medical devices, no parallel advice is contemplated with regulators (44). However, not all manufacturers have access to European services, because the criteria to be selected include important cross-border activity, major EU-wide added value and alignment with the Union Clinical Research Priorities. Therefore, national advice services still play an important role in the process.

In terms of manufacturers receiving parallel advice at European level, the process was found to be positively accepted (30). Data suggests that even if manufacturers tend to comply with regulatory advice (8), pharmaceutical companies were increasingly interested in seeking advice from an HTA perspective. The main reason for this is to improve the efficiency of the studies, enable a better study design and support the goals for a positive HTA recommendation for reimbursement (45).

However, manufacturers are aware that there are many areas where the regulator and payer/HTA are misaligned. After analysing the advice received by multinational companies in different countries, companies were actively taking advice and incorporating HTA requirements into their development process. That said, certain challenges related to divergencies found in the HTA methods and the decision-making process across different jurisdictions have been identified. In the results published in 2014 with data from Australia, Canada, England, France, Germany, Italy, Spain and the USA, HTA requirements incorporated by companies were related to patient-reported outcomes (84%), the endpoints that were acceptable for HTA (74%), and cost-effectiveness analysis (74 %) (45). In a more recent analysis, the authors found that HTA requirements were considered and implemented in the evidence generation plan in 63% of cases. In this analysis, information obtained between 2014 and 2018 from the same countries was considered. However, practices varied between companies, ranging from 37% to 100% of the developed products, showing different strategies among the participating companies. In that period, the most commonly accepted technical HTA requirements among the 65 included products were safety measures (92%), secondary endpoints acceptable for HTA (89%), patient selection criteria (88%), study design issues (88%), primary endpoints acceptable for HTA (86%) and duration of the trials (85%) (46).

Manufacturers have more frequently used single HTA agency advice, particularly from G-BA and NICE, although SA was the most influential in development programs (45).

Manufacturers have also stated that whereas EUnetHTA provided an overview of the opinions of HTA, the advice was not as in-depth as that provided by individual national advice services (20).

Pharmaceutical manufacturers have identified other benefits of the process, such as the reduction of the risk of the development programs and the creation of a common understanding of unmet medical needs (31).

However, the value of any dialogue depends on the stability of the advice or the time when it is provided (31). And, as has been previously stated, no data on the impact of ED/SA processes on medical devices have been published.

In this review, not many examples or services have been identified for medical devices and diagnostics. This is relevant as the development of “Medtech” technologies is more challenging. These technologies are less regulated and structured than those of pharmaceuticals. For instance, no parallel advice with regulatory agencies has been identified for medical devices or publications on the impact of SA on development and final reimbursement decisions. Nevertheless, in the European HTA regulation, it is mentioned that parallel advice for medical devices may take place with the consultation of expert panels in accordance with Article 61(2) of Regulation (EU) 2017/745 (44). In this systematic review, only three HTA organizations offered formal advice for non-drug technologies (32, 35, 37). Two of these use the META tool, the instrument developed by NICE to help find the gaps in evidence in the development of medical devices (32, 37). No studies on the opinion of manufacturers about the usefulness of the received advice have been published. It would be interesting to know what non-drug developers expect to obtain from HTA advice and if different options could be offered to them to improve their product development. As mentioned above, NICE offers different types of services to manufacturers and it could therefore be of interest to have further information on the experience and the issues that have arisen.

Although some authors have tried to measure the impact of advice on marketing authorization and the success of reimbursement, it may still be too early to assess the impact of those processes as time is needed from the ED until the final assessment, and some of the products that are in development get discontinued (29). Discouraging further developments could also be considered to have an impact.

In addition, not all manufacturers implement the advice given by HTA bodies. However, when advice is followed, the results of the clinical trials or research might not indicate the aptness of the product, so reimbursement may not be obtained. For example, in one study in France, of the 50 clinical trials that had been launched for medicinal products that received advice from HAS, 10 obtained negative results which did not support the use of the product, and 29 trials were still ongoing (12), so it was difficult to measure the impact of the given advice on the process.

The type of technology being considered, the stage of development or its complexity could determine how exhaustive the advice could be. Face-to-face meetings may be required, thereby lengthening the process. Whether or not written advice reports are required, even if they are widely considered to increase confidence in the process, will have an impact on the schedule (30). For example, NICE produces formal written post-consultation reports, whereas in Sweden the responsibility for documenting any discussion lies with the applicants themselves (30).

The time and resources required for engagement processes have to be considered, not only by the HTA bodies but also manufacturers, especially if engagement has or will have little impact on HTA final recommendations about the product (3).

Another important challenge in relation to engagement is the changing nature of HTA “*from a reactive to a pro-active process*” (3). In the study about the opinion of manufacturers on the SA offered by the German G-BA, even though a positive trend in the industry's perception of the SA received was observed over time, more active involvement of the G-BA professionals was identified as one of the factors that could improve the quality of the SA offered (28).

The nature of the advice given by all HTA organizations is non-binding. This issue could be considered by manufacturers either as an obstacle or an advantage (20). At the same time, the fact that public organizations could advise private companies also poses ethical challenges such as a conflict of interests. In HAS, besides the obligation to maintain professional secrecy and ensure the absence of a conflict of interests, HAS staff participating in EDs are not allowed to take part in future assessment and appraisal processes. Notwithstanding, professional secrecy is also asked of experts and patients who may be involved in ED processes (34).

It is considered increasingly important to include other stakeholders in the process, as is the case of patients or health care providers (3). The ED of EUnetHTA between June 2017 and May 2021 involved patients in 85% of the ED procedures (30). In addition, regulatory and HTA parallel advice has also been one of the proposed services offered by EUnetHTA. Even though ED processes started with EMA as an observer, the EUnetHTA ED process was improved during the project period because of the feedback received from all the stakeholders involved (HTA bodies, EMA, HTDs, and patients). This led to the simplification of the internal processes and to the acceleration of the ED process itself (31). The problem is that nowadays there is not a stable offer regarding this joint advice service, and how this service will be offered in the future is still to be defined.

Finally, HTA ED should not only focus on questions posed by the industry for specific products. In the HTAi Policy Forum meeting “*Changing HTA Paradigms”*, it was noted that scientific dialogue could also be focused on classes of products and/or disease areas, with the aim, for example, of validating new endpoints for new treatment approaches (3). These types of documents would be useful for manufacturers to focus their product development on what is needed for the management of a specific disease/s. One example would be the pilot programme that was developed to launch recommendations on the selection criteria, interventions, comparators, etc. to be used in Phase 3 or 4 clinical trials that were designed to assess the value of pharmaceutical therapies in mild or moderate Alzheimer's disease (47). Galbraith et al. (31) also suggested that to be more efficient, HTA could anticipate those areas where advice could be given for several technologies aimed at the same indication at the same time, offering indication-specific advice (31).

## Strengths and limitations

Although some earlier reviews have been published in relation to this topic, this is the first systematic review that combines both a systematic search for articles describing ED and SA services offered by HTA bodies as well as an active search for information in gray literature (i.e., HTA websites).

Apart from identifying processes and timelines, the study also examined (when available) the cost of scientific advice offered by different organizations, and information which is not always easy to identify, but which could be useful for companies and researchers.

From the analysis of the selected services, it is apparent that there is a lack of EA for Medtech in comparison to pharmaceutical entities. The process and outputs for those advice services also varies greatly among different organizations. Also, parallel processes that involve not just HTA bodies but also Regulatory Agencies pose a challenge.

It should be noted that it was not possible to obtain all the required or desired information for all the HTA organizations described above so the data or information presented on HTA organizations varies. The confidential nature of the outcome or result of advice services has also made it difficult to compare the type of advice given by each HTA body and, therefore, analyse its possible impact on the evidence generation plans for product development. While measuring this impact is itself a challenge, intermediary results could be considered, for example, the quality of the advice given in the final assessment or appraisal result should the value of the product not be demonstrated in the clinical studies. ED should not be bound by a positive reimbursement decision, it should just ensure that the evidence is suitable for informed reimbursement/coverage decisions, or what is more, continue encouraging or discouraging the development of the technology by innovators and companies. The result of such a decision cannot be predetermined by an ED.

## Considerations and future directions

In conclusion, this systematic review summarizes the main initiatives in relation to the advice given by HTA bodies to international manufacturers, highlighting the absence of standard definitions and the need to define the types of services that HTA could offer to companies, depending on the type of technology or the stage of development, among other factors. In this regard, few examples or services for medical devices and diagnostics have been identified, in comparison to the drug sector. The possibility of requesting parallel advice involving the Regulatory Agency is welcomed by manufacturers, although a clearer definition about what to expect from those services would be important for HTA bodies, regulators and manufacturers. There is still work to be carried out on developing the best advice services, and in the coming years, the implementation of the new European HTA regulation will help establish services that are better aligned to the needs of manufacturers and health care services and other stakeholders, thereby making the service more effective and efficient.

Now is the time to define the advice services that could be offered by HTA bodies to health technology developers to ultimately provide safe, effective and affordable health services to patients.

## Data availability statement

The original contributions presented in the study are included in the article/Supplementary material, further inquiries can be directed to the corresponding author/s.

## Author contributions

LG-C designed the search strategy. LG-C, NI-R, GB-A, KC-A, MG-S, and ID participated in the screening and final study selection. IG-I participated as third researcher to discuss and resolve any discrepancies. NI-R and LG-C participated in the data extraction and analysis. NI-R wrote the draft of the manuscript in collaboration with LG-C and IG-I. MS, AP-P, HD, and MO reviewed and commented the manuscript. All authors conceived, designed the content of the article, reviewed, and approved the final version of the manuscript.

## Funding

The contents of this paper arise from the project 814607/*SAFE*-*N*-*MEDTECH* which has received funding from the European Union's Horizon 2020 research and innovation program. The responsibility for its contents lies with the author(s), and neither the *SAFE*-*N*-*MEDTECH* Coordinator, the European Commission, nor any other body of the European Union is responsible for any use that may be made of the information contained therein.

## Conflict of interest

Authors KC-A, MG-S, EL-P, ID, CG-P, AP-P, and MS were employed by Keralty SAS. The remaining authors declare that the research was conducted in the absence of any commercial or financial relationships that could be construed as a potential conflict of interest.

## Publisher's note

All claims expressed in this article are solely those of the authors and do not necessarily represent those of their affiliated organizations, or those of the publisher, the editors and the reviewers. Any product that may be evaluated in this article, or claim that may be made by its manufacturer, is not guaranteed or endorsed by the publisher.
