# Supplementary material for: A systematic review of the early dialogue frameworks used within health technology assessment and their actual adoption from HTA agencies
Source: Front Public Health. 2022 Oct 6;10:942230. doi: 10.3389/fpubh.2022.942230 (PMC9583828; doi:10.3389/fpubh.2022.942230)
Supplement: Supplementary file 1 [file Data_Sheet_1.docx]

**Supplemental Material 1: Search Strategy**

**Search Strategy**

Databases: Pubmed, Embase, Scopus, Web of Science

Search date: October 2020

Limits: Search fields: title/abstract or subject

#1 "health technology" OR "technology assessment" OR "policy making" OR "health policy" OR "decision making" OR regulatory OR regulation

#2 "scientific advice" OR "early dialogue" OR "early advice"

#3 #1 AND #2

Results Pubmed 111

Embase 206

Scopus 402

WoS 312

**Supplemental Material 2: Excluded studies**

**Table of excluded studies**

|  | **Article** | **Reason** |
| --- | --- | --- |
| Id17 | Collignon et al. 2018 | Regulatory |
| Id20 | Balaisyte et al. 2018 | EA experiences: questionnaire |
| Id26 | Kondo et al. 2018 | No framework description |
| Id30 | O'Dwyer et al. 2017 | Regulatory |
| Id36 | Kondo et al. 2017 | Case study |
| Id55 | Schell-Inderst et al. 2015 | Not ED/EA/SA |
| Id58 | Hofer et al. 2015 | Regulatory |
| Id85 | Backhouse et al. 2011 | EA experiences: questionnaire |
| Id91 | Putzeist et al. 2011 | Regulatory |
| Id149 | Cieply et al. 2019 | Regulatory |
| Id168 | Poole et al. 2018 | Not framework description |
| Id187 | Dintsios et al. 2014 | Similar to other included study |
| Id212 | Hanna et al. 2016 | Case study |
| Id224 | Laricchia-Robbio et al. 2015 | Not ED/EA/SA |
| Id235 | Wang 2014 | Not specific framework description |
| Id336 | Matsuda 2019 | Regulatory |
| Id338 | Igbinovia et al. 2019 | Policy making |
| Id378 | Knottnerus et al. 2017 | Type of evidence |
| Id401 | Dzau 2016 | Case study |
| Id409 | Siyanbola et al. 2016 | Policy making |
| Id411 | Quirion et al. 2016 | Policy making |
| Id421 | Leyens et al. 2015 | Regulatory |
| Id423 | Dallmann 2014 | Type of evidence |
| Id438 | Klein 2013 | Type of evidence |
| Id442 | Raymond et al. 2013 | Type of evidence |
| Id445 | Sarría-Santamera et al. 2013 | Policy making |
| Id450 | Vamvakas 2013 | Type of evidence |
| Id459 | Waigmann et al. 2012 | No related content |
| Id467 | Pfleiderer 2012 | Type of evidence |
| Id477 | Marchand et al. 2011 | Case study |
| Id480 | Fears et al. 2011 | Type of evidence |
| Id481 | Lentsch 2011 | Type of evidence |
| Id644 | Hofer et al. 2018 | Regulatory |
| Id718 | Hofer et al. 2015 | Type of evidence |

References of excluded studies

- Collignon O, Koenig F, Koch A, Hemmings RJ, Pétavy F, Saint-Raymond A, Papaluca-Amati M, Posch M. Adaptive designs in clinical trials: from scientific advice to marketing authorisation to the European Medicine Agency. Trials. 2018 Nov 20;19(1):642. doi: 10.1186/s13063-018-3012-x.
- Balaisyte L, Joos A, Hiligsmann M. Early dialogue in Europe: perspectives on value, challenges, and continuing evolution. Int J Technol Assess Health Care 2018 Jan;34(5):514-8. doi: 10.1017/S0266462318000545.
- Kondo H, Saint-Raymond A, Yasuda N. What to Know About Medicines With New Active Ingredients Approved in FY 2016 / 2016 in Japan and EU: A Brief Comparison of New Medicines Approved in Japan and the EU in 2016. Ther Innov Regul Sci 2018 Mar;52(2):214-9. doi: 10.1177/2168479017720248.
- O'Dwyer L, Nolan L, Fisher C. Supporting Innovation through Regulation and Science: Ireland as an Innovation Hub for Health Products. Biomed Hub 2017 Nov;2(Suppl 1):222-9. doi: 10.1159/000481427.
- Kondo H, Sugita T, Ida N, Fukushima H, Yasuda N. A Comparison of PMDA and EMA Consultations for Regulatory and Scientific Matters in Drugs and Regenerative Medicine Products. Ther Innov Regul Sci 2017 May;51(3):355-9. doi: 10.1177/2168479016680259.
- Schnell-Inderst P, Mayer J, Lauterberg J, Hunger T, Arvandi M, Conrads-Frank A, et al. Health technology assessment of medical devices: What is different? An overview of three European projects. Z Evid Fortbild Qual Gesundhwes 2015;109(4-5):309-18. doi: 10.1016/j.zefq.2015.06.011.
- Hofer MP, Jakobsson C, Zafiropoulos N, Vamvakas S, Vetter T, Regnstrom J, Hemmings RJ. Regulatory watch: Impact of scientific advice from the European Medicines Agency. Nat Rev Drug Discov. 2015 May;14(5):302-3. doi: 10.1038/nrd4621.
- Backhouse ME, Wonder M, Hornby E, Kilburg A, Drummond M, Mayer FK. Early dialogue between the developers of new technologies and pricing and reimbursement agencies: a pilot study. Value Health 2011 Jun;14(4):608-15. doi: 10.1016/j.jval.2010.11.011.
- Putzeist M, Mantel-Teeuwisse AK, Gispen-de Wied CC, Hoes AW, Leufkens HG. Regulatory scientific advice in drug development: does company size make a difference? Eur J Clin Pharmacol 2011 Feb;67(2):157-64. doi: 10.1007/s00228-010-0919-x.
- Cieply B, Macaulay R, Sutcliffe D, Wang G. PNS128 Ready for prime time? Have EMA prime designations supported accelerated regulatory approval and patient access? Value in Health. 2019;22:S783. doi: 10.1016/j.jval.2019.09.2029.
- Poole RL, Jarrom D, Myles S. #24 Health Technology Wales - Assessing Value, Optimising Use (poster presentation). International Journal of Surgery 2018 Nov;Conference(Supplement 1):S7. doi: 10.1016/j.ijsu.2018.10.025.
- Dintsios, C., & Schlenkrich, S. VP107 Pharmaceutical Industry's Experiences with the German Health Technology Assessment Scientific Advice. Int J Technol Assess Health Care. 2017, 33(S1): 197-8. doi: <https://doi.org/10.1017/S026646231700366X>
- Hanna E, Azaiez C, Auquier P, Toumi M. A new accelerated early access process for diagnostics in France. Value in Health 2016 Nov;Conference(7):A706. doi: <https://doi.org/10.1016/j.jval.2016.09.2064>
- Laricchia-Robbio L, Rico L, Oruezabal RI, Del CC, Carmona G, Mata R, et al. The Andalusian initiative for advanced therapies: A pioneer model to foster the incorporation of these innovative therapies into the clinical practice. Cytotherapy 2015 Jun;Conference(var.pagings):S80. doi: <https://doi.org/10.1016/j.jcyt.2015.03.584>
- Wang T. Benchmarking the Impact of HTA on New Medicines Development and Coverage Decision Making. Value Health. 2014 Nov;17(7):A798. doi: 10.1016/j.jval.2014.08.478.
- Matsuda Y. Global Regulatory Landscape. AAPS PharmSciTech. 2018 Dec 17;20(1):2.
- Igbinovia FO, Krupka J. An overview of science, technology, and innovation (STI) policy for driving a manufactured-led economic and social transformation of Nigeria. 2019 p. 9553-66.
- Knottnerus JA, Tugwell P. Methodology of the 'craft' of scientific advice for policy and practice. J Clin Epidemiol. 2017 Feb;82:1-3. doi: <https://doi.org/10.1016/j.jclinepi.2017.01.005>
- Dzau VJ. The Institute of Medicine: ensuring integrity and independence in scientific advice on health. Lancet. 2016 Apr 16;387(10028):1686-92. doi: 10.1016/S0140-6736(15)00468-7.
- Siyanbola, W., Adeyeye, A., Olaopa, O. et al. Science, technology and innovation indicators in policy-making: the Nigerian experience. Palgrave Commun 2, 16015 (2016). <https://doi.org/10.1057/palcomms.2016.15>
- Quirion, R., Carty, A., Dufour, P. et al. Reflections on science advisory systems in Canada. Palgrave Commun 2, 16048 (2016). <https://doi.org/10.1057/palcomms.2016.48>
- Leyens L, Richer É, Melien Ø, Ballensiefen W, Brand A. Available Tools to Facilitate Early Patient Access to Medicines in the EU and the USA: Analysis of Conditional Approvals and the Implications for Personalized Medicine. Public Health Genomics. 2015;18(5):249-59. doi: 10.1159/000437137.
- Dallmann G. Regulatory Review: Clinical to Market Transition. Handbook of Therapeutic Antibodies: Second Edition. 2-4 ed. 2014. p. 1263-82.
- Klein, A. The khat ban in the UK What about the ‘scientific’ evidence? Anthropology Today, 2013. 29 (5). pp. 6-8. <https://doi.org/10.1111/1467-8322.12057>
- Raymond AS, Humphreys AJ. Human Medicinal Products in the European Union: Regulations, Directives and Structures. The Textbook of Pharmaceutical Medicine2013. p. 360-78.
- Sarría-Santamera A, Schoten EJ, Coenen TM, Gunning-Schepers LJ, Pauwels A, Allander SV, Wysocki MJ, Ciutan M, Segovia C. A framework for scientific advice on health: EuSANH's principles and guidelines. Health Res Policy Syst. 2013 Feb 22;11(1):6. doi: 10.1186/1478-4505-11-6.
- Marchant GE, White A. An international nanoscience advisory board to improve and harmonize nanotechnology oversight. Journal of Nanoparticle Research. 2011;13:1489-98. doi: 10.1007/s11051-011-0226-1
- Vamvakas S. Regulatory Experience of Biomarker Qualification in the EMA. The Path from Biomarker Discovery to Regulatory Qualification. 2013. p. 35-40.
- Waigmann E, Paoletti C, Davies H, Perry J, Kärenlampi S, Kuiper H. Risk assessment of Genetically Modified Organisms (GMOs). EFSA Journal 2012;10(10). <https://doi.org/10.2903/j.efsa.2012.s1008>
- Pfleiderer M. The bumpy road toward vaccine registration: How to overcome regulatory hurdles. Development of Novel Vaccines: Skills, Knowledge and Translational Technologies. 2012. p. 287-300. doi: 10.1007/978-3-7091-0709-6_11
- Fears R, ter Meulen V. European Academies Science Advisory Council (EASAC). The Politics of Scientific Advice: Institutional Design for Quality Assurance. 2011. p. 342-52.
- Lentsch J, Weingart P. Introduction: The quest for quality as a challenge to scientific policy advice: An overdue debate? The Politics of Scientific Advice: Institutional Design for Quality Assurance. 2011. p. 3-18. doi: 10.1017/CBO9780511777141.001
- Hofer MP, Hedman H, Mavris M, Koenig F, Vetter T, Posch M, Vamvakas S, Regnstrom J, Aarum S. Marketing authorisation of orphan medicines in Europe from 2000 to 2013. Drug Discov Today. 2018 Feb;23(2):424-433. doi: 10.1016/j.drudis.2017.10.012.
- Hofer MP, Jakobsson C, Zafiropoulos N, Vamvakas S, Vetter T, Regnstrom J, Hemmings RJ. Regulatory watch: Impact of scientific advice from the European Medicines Agency. Nat Rev Drug Discov. 2015 May;14(5):302-3. doi: 10.1038/nrd4621.

**Supplemental material 3: Description of included studies**

**Systematic or narrative reviews (articles and conference abstracts)**

| **Reference** | **Objective** | **Organization/s mentioned** | **Type of technology** | **Material and methods** | **Results (type of procedures, impact assessment etc…)** | **Terms used to describe the process** | **Other considerations** |
| --- | --- | --- | --- | --- | --- | --- | --- |
| Cuche 2014 (id 59) | To present and evaluate the different options available for early HTA consultation (during drug development/Phase III) in the major European markets from the industry perspective. | G-BA/IQWiG; TLV, NICE, CVZ, EuneHTA, EMA. | Drugs | - Web sites of the major national-level HTA bodies in Europe searched. - EMA and EuneHTA web sites for information on pan-European HTA processes. - Pragmatic review of specific examples of companies publishing their early HTA consultative efforts. | Eight HTA bodies identified that met selection criteria, among four main models for companies seeking early consultative HTA advice:  1. Single market HTA advice: NICE as example.  2. Single-market parallel regulatory and HTA advice: G-BA/IQWiG with BfArm (he Federal Institute for Drugs and Medical Devices).  3. Multi-country HTA advice: EUnetHTA Joint Action 2 Pilot 15/16  4. Parallel regulatory and multi-country HTA advice: EMA Pilots (EMA’s regulatory scientific advice procedure in parallel with HTA bodies advice (Ref 17;18) | Early advice consultation with HTA bodies. | Regional HTA agencies, or HTA agencies from countries with fewer than six million inhabitants, were not included within this assessment. |
| Ciani 2014  (id 63) | To describe current HTA activities with a potential impact throughout the drug development process of pharmaceuticals, with a comparative emphasis on the systems in place in Italy and in the UK. | NICE  AIFA  (bi or tripartite early dialogue between manufacturers, regulators and HTA asessors). | Drugs | Extensive literature and website review | Three major classes of HTA activities, beyond mainstream HTA, with the potential to influence the drug development program: 1) horizon scanning and early HTA; 2) bipartite and tripartite early dialogue between manufacturers, regulators, and HTA assessors; and 3) managed market entry agreements. | Early dialogue  Scientific advice from HTA | Article.  From early stages of clinical research up to postauthorization studies, there is a trend toward increased collaboration between parties, anticipation of market access evidence collection, and postmarketing risk-sharing.  Heterogeneity of HTA practices increases the complexity of the market access environment., There are signals that market access departments are gaining importance in the pharmaceutical companies, but there is still a lack of evidence and reporting on how the increasing relevance of HTA has reshaped the way clinical development is designed and managed. |
| Palkments 2017  (id 189) | To understand early Health Technology Assessment (HTA) advice opportunities in European countries: to characterise the scope of advice offered (e.g. formal vs. informal, written vs. oral, separate HTA advice or joint with regulatory scientific advice), the early advice process, and costs associated with advice. | Some mentioned: NICE, G-BA, AIFA, HAS, TLV…Also EUnetHTA and countries like Belgium, Norway, | Not mentioned | A structured literature search was conducted incorporating National HTA websites, HTA conference websites and PubMed. The country scope included fifteen European countries: Austria, Belgium, Denmark, Finland, France, Germany, Hungary, Ireland, Italy, Netherlands, Norway, Poland, Spain, Sweden, and United Kingdom | Formal national early HTA advice (separate from regulatory advice and with the official description of the process and application form) is offered in Germany, United Kingdom, France and Italy. Sweden, Norway and Belgium have a procedure allowing manufacturers to obtain answers to HTA-related questions while applied for national early scientific regulatory advice. The majority of countries participate in multi-stakeholder programs: parallel EMA-HTA scientific advice procedures, the EUnetHTA early dialogue and Shaping European Early Dialogues projects. Most agencies recommend that early advice is sought between phase II and phase III of clinical development. Consultation times vary from 6-8 weeks in Norway to 18 weeks in France and United Kingdom. Advice is usually provided through hearings (teleconferences and face-to-face meetings) and some agencies like G-BA (Germany), AIFA (Italy), HAS (France), TLV (Sweden) and NICE (UK) provide a written report. There is no fee in Sweden, Belgium and Norway, while in all other countries it varies from €2,000 - £50,000. In France the early dialogue is free of charge, however, the advice is given only for innovative products. | HTA early advice.  Regulatory advice.  Parallel EMA-HTA scientific advice procedures.  The EUnetHTA early dialogue. | Conference abstract.  The scope of HTA early advice varies significantly across geographies as do the costs and consultation times for advice. |
| Vlachaki 2017  (Id 193) | To compare the three most widely used single early SA  processes offered NICE, UK, HAS, France, and G-BA, Germany | NICE  HAS  G-BA | Drugs and devices | This overview and suggestions are based on review of NICE, HAS and G-BA SA processes and requirements as well as authors’ expertise. Authors reviewed the SA processes, timelines, template availability, and role of each participating party. | SA timelines vary from 2 to 5 months.    NICE and HAS provide  briefing book templates, while G-BA provides a request form.  NICE and HAS evaluate cost-effectiveness, while G-BA only discusses clinical benefit.    Only NICE involves patient representatives in the SA process.  Fees vary from nothing to £50,000.  The advice is not legally binding for any of the processes; however, the received guidance provides valuable insights on the **choice of comparators, endpoints, study design and health economics.**    SA is country and payer specific and helps to build relationships between all parties.    The SA process helps to align the company’s position and involve all internal stakeholders at an early stage. | Early scientific advice | Congress abstract.  In addition to aiding in the optimisation of clinical development programmes to generate evidence that is most relevant to HTA bodies, regulators, payers and patients, HTA SA dialogue can provide insights into country specific issues. Furthermore, the SA process helps to align the company’s position and involve all internal stakeholders at an early stage. |
| Craddy P 2018  (Id 167) | Review?  The aim of this study is to compare the key aspects companies must consider when seeking payer scientific advice | -NICE  -G-BA  -HAS  -AIFA  -Parallel consultation with EMA and HTA bodies. |  | Secondary research was conducted to identify the payer scientific advice processes at NICE, G-BA, HAS, AIFA and the parallel consultation with the EMA and HTA bodies. The timelines, submission requirements, scope of advice and fees for each procedure were then compared and contrasted. | Major differences between payer scientific advice procedures were identified.  The G-BA and NICE’s Office of Market Access have the shortest timelines (advice within 10- 14 weeks).  NICE’s scientific advice and EMA’s parallel consultation takes 24 to 34 weeks.  The G-BA require the fewest submission materials; in contrast, the EMA, NICE, HAS and AIFA require a comprehensive briefing book, list of questions and company position for each question to be submitted prior to the meetings.  The G-BA provides relatively defined feedback, such as likely comparators or patient populations, whereas NICE also provides feedback on statistical analysis and health economic considerations.  The EMA parallel consultation also offers regulatory guidance but may not provide the depth of feedback compared with individual HTA consultations.  The cost of seeking advice varies considerably; HAS does not request fees, whereas the EMA parallel consultation incorporates fees from the EMA and each HTA involved in the parallel consultation, can exceed 50,000 euros. | Payer Scientific advice (to optimise their clinical trial designs from an HTA perspective). | Conference abstract.  Conclusion: variability regarding the scientific advice submission processes and scope of advice provided across the EU.  Pharmaceutical companies should give careful consideration as to which procedure is most appropriate for their needs, taking into account their timelines and resources and the type of payer insights required to optimise their clinical trial designs from an HTA perspective. |
| Gannedahl 2018  (Id 165) | To provide a review of the latest, as well as a future perspective on, developments with respect to accelerated access of medicines in the European Union with a particular focus on procedures for formal scientific advice. | Regulatory and HTA bodies’ parallel consultation (scientific advice) | Drugs for high unmet health needs. | Narrative review. No methodology stated. | Three different forms in Europe for parallel consultation scientific advice:  1) with regulatory agencies only (at national level, as BfArm in Germany, MHRA in UK, MPA in Sweeden or on a European level by EMA, through the Scientific Advice Working Party, named SAWP)).  2) with HTA bodies only (as a single country HTA-scientific advice or as multi-country engagement.  3) through integrated parallel regulatory HTA scientific advice with both regulatory agencies and HTA bodies, that could be at a:  - National level: as NICE and MHRA in England (such as EAMS or Joint Regulatory HTA advice), TLV and MPA in Sweden and G-BA and BfArM in Germany, or as parallel consultation between the EMA and HTA bodies.  - Pan-european level: EMA and the EuneHTA (termed parallel consultation). | Parallel scientific advice  Parallel consultation  Regulatory advice  HTA scientific advice  Joint regulatory HTA advice | For the regulatory: The advice procedure aims to facilitate the MA procedure by reducing potential areas for objections by the EMA during the approval process, further evident form the addition of PRIME, where iterative scientific advice is an important tool.  The overall aim of seeking HTA scientific advice is to support pharmaceutical companies in designing an evidence generation plan which satisfies the requirements of an HTA reimbursement submission in order to reduce likelihood of any delays in patient access.  Rather than focusing on the benefits and risks of a medicine and usually within a highly controlled setting, as in regulatory advice, HTA bodies look at the clinical and economic value within a real-life setting of current treatments and service pathways. |
| Boss J 2019  (id 146) | In 2019, CADTH announced two parallel scientific advice (SA) programs: with NICE and with Health Canada.  **Aim**: to outline the processes for obtaining advice through these programs and identify the value to manufacturers. | CADTH/NICE  Health Canada | Not specified. | Information about CADTH’s parallel SA programs was obtained through public-domain sources, and through strategic discussion and collaboration with CADTH (abstract authors). Topics included similarities and differences between the programs and the rationale for CADTH’s collaboration with NICE. | CADTH’s parallel SA programs with NICE and Health Canada include submission of the **same briefing book** to each organization, a face-to-face meeting, and provision of separate advice reports. Slight differences: fee schedule, format/location of face-to-face meeting, timelines, and a summary of alignment which is provided by the CADTH-NICE program only.  The collaboration between CADTH and NICE allows for synergies between the standard SA programs of each HTA body (with inherent similarities). CADTH’s standard SA program differs from NICE **in the provision of advice**. During the face-to-face meeting with CADTH, manufacturers can discuss draft advice and raise points of clarification in real-time. This differs from NICE’s standard SA program where face to-face meetings tend to occur earlier in the process and are more exploratory in nature. | Parallel Scientific Advice (SA) | Conference abstract. |

**About individual organizations**

| **Reference** | **Objective** | **Organization/s mentioned** | **Type of technology** | **Material and methods** | **Results (type of procedures, impact assessment etc…)** | **Terms used to describe the process** | **Other considerations** |
| --- | --- | --- | --- | --- | --- | --- | --- |
| Rose M 2019  (id 154) | To establish whether methodological issues in evaluating advanced therapy medicinal products (ATMPs) identified by the Regenerative Medicine Expert Group (RMEG) mock technology appraisal are consistent with advisory experiences of NICE Scientific Advice (NICE SA). | NICE | Advanced therapy medicinal products (ATMS) | We undertook a qualitative and quantitative review of advice requested on ATMPs, by companies between 2011 and 2018, using our project database. Thematic data were extracted from company questions and then compared to issues identified by the RMEG mock appraisal conducted by NICE and the University of York (2015). | Twenty-five ATMP NICE SA projects were identified, with 15 gene and 10 cell therapies. The RMEG mock appraisal report identified the following key methodological issues: quantifying and presenting clinical outcomes, decision uncertainty based on immature data, discount rate methodology, and innovative payment mechanisms. All NICE SA advisory projects addressed questions on quantifying and presenting clinical outcomes. Of 25 projects, nine requested advice on surrogate outcomes and 19 requested advice on measuring and reporting health-related quality of life or patient-reported outcome measures. There was substantial evidence that companies were concerned about immature outcome data as 21 projects requested advice on trial duration, extrapolation, model time horizons, or observational and RWE to inform long-term outcomes. As highlighted in the ‘mock appraisal’, many NICE SA projects advised on single-arm trials, historical controls, and trials where patients act as their own control (n=16). Ten projects explored evidence synthesis methodology, including utilising individual patient and aggregate data from single-arm and comparative trials. Several projects sought methodological clarity in informing and constructing models for these potentially curative therapies. Few projects queried discount rates, innovative payment mechanisms or managed access agreements | NICE scientific advice | Conference abstract.  Conclusion: NICE SA ATMP experiences provide real-world validation of most issues identified in the RMEG mock appraisal. NICE SA provision of advice on these issues facilitates improvements in ATMP health technology assessments. |
| Maignen FM 2014  (id 236) | Regulatory Scientific Advice (SA) provided by EMA, FDA, MHRA and other agencies is highly demanded by manufacturers, but health technology assessment (HTA) scientific advice is still far from becoming a routine step in the product development cycle. NICE has been running an advisory service for 5.5 years | NICE | Medicinal products and 2 diagnostic tests | This work presents analysis of requests to the programme: types of advice projects, number and type of requests per company, clinical indication, stage of clinical development when the advice is sought, reason for seeking advice and current development and regulatory status of product | Between 2009 and 2014 NICE conducted 109 advisory projects (107 medicinal products and two diagnostic tests). 23 were done in parallel with regulatory agencies and/or other HTA bodies.  78% of all requests in the following four therapeutic areas: oncology, neurology, rheumatology and cardiology.  Majority of products (61%) were in phase II when advice was sought.  At the time of this analysis, 71 products (66%) still in development, 6 (5.5%) for a marketing authorisation review (MA), 8 (7.5%) received a MA, authorisation not granted to 2 products (2%) and clinical development discontinued in 20 cases (19%). Most products that received NICE scientific advice are yet to be referred to the technology appraisals programme. | HTA scientific advice. | Conference abstract.  Conclusion:  Over the last few years, requests for scientific advice diversified into personalised medicines, regenerative medicines and products for rare and very rare diseases. Most HTA scientific advice requests continue to come from top 20 Pharma companies, however we are starting to see an increasing number of inquiries and project bookings from small-medium size companies |
| Philips 2012 (id 256) | They present a detailed analysis of the NICE SA programme through 2009 to 2012. | NICE | Drugs and devices | The NICE SA process involves assessment of the manufacturer’s briefing book with input from external clinical experts, health economists and methodological experts. Following a face-to-face meeting between manufacturer, expert panel and SA technical team, a formal written report summarising the advice is produced.  NICE SA provides advice alongside the European Medicines Agency (EMA) and other Health Technology Assessment (HTA) agencies. Such collaborations do not include a formal written report, advice is given verbally at joint meetings. | To date the programme has successfully completed 52 formal written advice projects.  Requests for advice alongside the EMA and other HTA agencies have been increasing with 13 projects completed since 2010. Specifically, we will report on the types of questions posed in manufacturers’ briefing books including questions on health economic evaluation. We will include a breakdown by therapeutic area, frequency of requests by company, type of company, profiles of participants at meetings, and trends over time. | NICE Scientific advice programme (to provide written advice to pharmaceutical companies and device manufacturers about development plans for their products to ensure they produce relevant evidence for future submission to NICE. | Conference abstract |
| Maignen F 2017 (one common autor with Philips 2012)  (id 41) | The primary objective of the study was to analyse the proposed clinical development and economic evaluation plans for investigational medicinal products for which pharmaceutical companies have sought health technology assessment (HTA) scientific advice (SA) | NICE | Medicinal products  50% (87) were new chemical entities and 40% (68) biologicals (excluding ATMPs) (gene). Remaining products include 9 (5%) gene or cell ATMPs (6 of them developed by SMEs). | We have selected and analysed all the scientific advice procedures undertaken by National Institute for Health and Care Excellence (NICE) SA between 1 January 2009 and 3 December 2015 for investigational medicinal products. We have mapped the questions asked by the companies and the areas of advice highlighted in the advice reports to the sections of the NICE methods guide to the technology appraisals (2013). | An overwhelming proportion of SA procedures have addressed questions related to the clinical development and specifically the main pivotal efficacy studies. Approximately a quarter of the questions relate to the approaches to economic evaluation. Questions raised in European Medicines Agency/HTA procedures generally focus on **clinical efficacy issues** whereas cost-effectiveness ones tend to dominate in NICE only procedures. Our analysis shows that the issues mostly discussed **in the HTA SA are the choice of comparator, the generalisability of the clinical trial evidence to the NHS practice and the impact of the clinical trial outcomes on quality of life and survival.** Less disagreement with the developers’ plans was seen in the choice of clinical endpoints, population definition, position of the technology in the treatment pathway and study design. | Scientific advice procedure (about clinical development and specifically pivotal efficacy studies). 25% of the questions relate to economic evaluation. | Article  Scientific advice is designed to improve the quality of evidence and approaches to evidence generation for future regulatory approval and HTA evaluation. Our experience to date suggests that payer requirements are inconsistently integrated in the clinical development programmes. More efforts should be dedicated to demonstrating the clinical value of new medicinal products to patients and key decisionmakers |
| Heyes A 2019 (Id 145) | This review compares HTA ED processes offered by EUnetHTA and NICE, provides information on good quality interactions for manufacturers, and describes new service options introduced by NICE, including contingency plans should the UK leave the European Union (EU) | NICE (and others: EuneHTA  EMA  CADHT  Blue Cross Blue Shield (United States)  FINOSE (Norway, Sweden, Finland). | Pharmaceutical products. | A targeted review of literature and Internet-based sources identified the most recent information on ED processes provided by NICE and EUnetHTA. Both offer joint regulatory and HTA advice; this review focused on the HTA advice process for pharmaceutical products. | NICE services:  **1. EUnetHTA ED process**: involves the ED Working Party (EDWP) with agencies from France, Germany, Hungary, Italy, UK, Belgium, and The Netherlands; additional HTA bodies may participate. The final group of HTA bodies participating in a specific ED will comprise the ED Committee (EDC).  • Manufacturers submit a briefing book with product and disease information plus specific questions on clinical and economic issues for HTA. EUnetHTA and NICE include the opportunity for face-to-face discussion and written recommendations.  **2.Standard advice**  **3. Express advice**  **4. PRIMA:** service specifically at the economic model (**Preliminary Independent Model Advice**), providing expert independent advice to improve the quality of health economic models. Includes independent expert advice, identification of any flaws in structure or coding and advice on the transparency and usability of the model.  **5. Parallel Advice With Other Payers**: Canadian HTA (Canadian Agency for Drugs and Technologies in Health) to provide joint advice, with Blue Cross Blue Shield (United States) and FINOSE (Norway, Sweden, Finland).  **6. Brexit**  If NICE is part of a joint EUnetHTA ED, all existing contracts will be fulfilled, and NICE will provide its own advice letter and a NICE-specific face-to-face meeting.  Contingency plan in place to enable advice concurrently with European Medicines Agency (EMA) or EUnetHTA. | Early Dialogue  HTA advice  Advice | Conference Poster.  NICE's process to engage in early dialogue is particularly well and clearly defined with clear timelines and deliverables. |
| Myles S 2020 (Wales)  (id 136) | HTW is collaborating with the Bevan Commission through their national Health Technology Exemplars (HTEs), which partners NHS and industry stakeholders to strengthen innovation within the Welshhealth system. | HTW (Health Technology Wales) | Non-medicine technologies (medical devices, diagnostics or procedures). | Health technology assessment (HTA) methods were used to produce topic exploration reports for assessing the evidence underpinning applicant innovations.  A“Dragons’Den” expert panel was convened to select the successful HTEs (Health technology Exemplaires) | Fourteen Bevan HTEs were awarded funds, which were matched by industry partners. Application of HTA methods resulted in more critical consideration of technology value propositions, including: developing pull models of innovation focused on delivering health technology solutions for current problems facing NHS Wales; supporting early dialogue between the NHS and industry partners around demonstrating evidence of improved patient outcomes; and focusing on transformative rather than incremental innovation. | Early dialogue between NHS and industry | HTW has a Scientific Advice program for non-medicine technologies (available at: <https://healthtechnology.wales/sas/#:~:text=The%20HTW%20Scientific%20Advice%20Service,providers%2C%20patients%20and%20service%20users>. ). |
| Fernández 2019 (id 135) | To provide a first overview of clinical develop-ments for which pharmaceutical companies sought an early dialogue with HAS. | HAS (with EMA and/or others European HTA bodies) | Drugs.  Requests are eligible when the product has a new mechanism of action, if there is an unmet or partially met medical need in the  claimed indication and when the pivotal study has not yet started. | For each product that went through an early dialogue procedure with HAS, information regarding the clinical development was collected on pharmaceuticals companies’pipelines,clinicaltrials.gov, the website of the European Medicine Agency(EMA) and HAS’s internal database. | By the end of 2018, HAS has performed 84 early dialogues (53 in collaboration with the EMA and/or others European HTA bodies).  Mainly focused on phase III trials.  Following early dialogue, the clinical study for which the company sought advice was not yet implemented in 25 cases.  When the clinical trial was effectively launched, results were negative in 10 cases, positive in 11 and still ongoing for 29. In nine cases, the clinical development was officially withdrawn or suspended before the initiation of the trial. Overall, only eight medicinal products were appraised by HAS, they all obtained a clinical added value score. | Early advice on evidence generation plans.  Early dialogue. | Conference abstract.  The success rate of clinical development for products that underwent an early dialogue procedure tends to be higher than data from literature, although it is likely to decrease in follow-up analysis. This could be partially explained by HAS’s eligibility criteria that **restrict early dialogues to promising products and by the scientific recommendations provided to pharmaceuticals companies.** |
| Wonder M 2013 (Australia)  (id 68) | To explore the practicality, feasibility, and sustainability of means of obtaining simultaneous scientific advice from both a regulatory and reimbursement perspective. | Tripartite early scientific advice: a pharmaceutical company (developer), the Therapeutic Goods Administration (TGA: regulator) and the Pharmaceutical Benefit Advisory Committee (PBAC) Secretariat (HTA agency) in Australia | New medicines | Advice was sought for two development compounds in different disease areas. **The focus was on matters of common interest to the TGA and the** **PBAC (i.e. the clinical evidence)**. Briefing books were prepared by the developer and supplied eight weeks prior to the meeting and only **verbal advice** was provided. | The pilot meeting took place in 2009. Each session lasted for approximately two hours and was structured around the questions in the briefing books. The representatives from the TGA and PBAC Secretariat provided well-informed, considered and careful advice for both compounds, which was predominantly actionable and practical.  Each briefing book included questions for the agencies aimed at testing the suitability of the proposed evidence plans for the purposes of supporting payer as well as regulatory decisions. | **Early Scientific Advice**  (**focused on proposed target patient population(s), indication(s), comparator (s), trial outcomes, and duration of follow-up of the proposed phase 3 clinical trials and how they had been determined.** | Briefing book for each compound outlining:  1) a proposed clinical development program and  2) submit it to the agencies 8 weeks in advance of a face-to-face meeting. |
| Plaud 2017 (Germany and France)  (Id 190) | To define what are the key concepts for assessment of innovative devices and how do they reflect inherent characteristics of medical devices | Institute for Quality and Economic Efficiency  (IQWiG) in Germany and the medical devices evaluation committee (CNEDiMTS) in  France. (Commission nationale d’évaluation des dispositifs médicaux et des technologies de santé) | Innovative **medical devices** | Due to the inherent characteristics of medical devices in terms of complexity, learning curve, life-cycle and regulation, determining the benefit of an innovative technology for patient care at the earliest stages might be difficult. Methodology for evaluating innovative medical devices by Institute for Quality and Economic Efficiency (IQWiG) in Germany and the medical devices evaluation committee (CNEDiMTS) in France has been analyzed in a comparative way taking into account key concepts of “innovation”, “benefit”, and “ efficiency”. | Both countries developed different approaches to bypass the evidence gap inherent to early developmental stage of a device in order to ensure patients fast access to safe and effective medical innovation. In Germany a testing regulation and early dialogue framework have been progressively developed to generate evidence along with the device manufacturer. In France, a fast-track for innovative devices has been designed to accelerate their market access and gain reliable technical data on added benefit for patients. Establishing an innovation pathway at European level based on patient registries to collect uniform data would make valuable contributions to the evaluation and monitoring of such disruptive innovation at early stage. | Early dialogue framework (to generate evidence along with the device manufacturer). | Conference Abstract.  Development of medical devices is an iterative process and differs from pharmaceuticals. Assessment of innovative technologies should take these specificities into account when assessing added benefit for patients. On that basis, both countries progressively strengthened their legal framework. However in practice it still takes time for innovation to reach the market and be reimbursed |
| Dintsios CM 2018  (Id 356)  (Previously published as Dintsios C (Charalabos)  2017; Germany)  (id 187) | Optional scientific advice (SA) for the early benefit assessment of pharmaceuticals is offered by the German decision maker, the Federal Joint Committee (FJC). The aim of this study was to elicit manufacturers’ experiences with the SA procedures offered by the FJC to date. | Federal Joint Committee (FJC) | Drugs | A preliminary survey on a small sample size was conducted. Subsequently, a questionnaire comprising eight items, which was developed on the basis of that survey, was used. Data were analyzed using qualitative and quantitative approaches | The elicitation, including a sample of 25 percent of the completed advice, highlighted the following, regarding the process as well as to the content shortcomings of the SA procedures from an industrial perspective: inconsistencies, FJC’s lack of expertise in conducting clinical trials, partially incomplete answers. and a low willingness of the FJC to engage in dialogue with industry were criticized. On the other hand, the majority of respondents expressed a positive attitude concerning unambiguousness, completeness, traceability, discussion atmosphere, and the protocol of the advice. Early SA, before pivotal trials start, showed a significantly higher completeness compared with late SA with respect to endpoints and study duration. Within 4 years the quality of FJC’s propositions on some topics improved significantly.  **The participating stakeholders within the scientific advice at the FJC and the timelines of the process.** Next to the FJC and the manufacturer representatives of the approval authorities are optionally and on request of the manufacturer involved as well. After the manufacturer submits the requirement form to the administrative office of the FJC the specialist counseling group of the FJC is performing literature searches to inform the working group for the early benefit assessment of pharmaceuticals of the FJC’s subcommittee for pharmaceuticals. This working group prepares the first version of the answers which are finalized by the subcommittee for pharmaceuticals. Subsequently the scientific advice takes place at the FJC where the manufacturers meet the representatives of the administrative office for explanatory responses. Finally, a written protocol is prepared by the administrative office (if necessary the subcommittee for pharmaceuticals is also involved) and forwarded to the manufacturer. | Scientific advice for the early benefit assessment. | Only a few statistically significant differences were detectable between early versus late SA. A positive trend in industry’s perception of the SA can be observed over  time. A more active involvement of additional stakeholders and the incorporation of procedural elements from other healthcare systems could improve the quality of the SA offered by the FJC.  The author, in addition to his academic affiliation, is employed by Bayer Vital GmbH. During the preparation of the manuscript, S.Sch. was a trainee at Bayer Vital GmbH. C.M.D. conducted the interviews, developed the questionnaire and prepared the manuscript. S.Sch. analyzed the data together with C.M.D. C.M.D. acts as the overall guarantor. |

**About EUneHTA/EMA (Alone or together).**

| **Reference** | **Objective** | **Organization/s mentioned** | **Type of technology** | **Material and methods** | **Results (type of procedures, impact assessment etc…)** | **Terms used to describe the process** | **Other considerations** |
| --- | --- | --- | --- | --- | --- | --- | --- |
| Harousseau 2015  (Id 222) | To describe de SEED project: a collaborative approach to EDs, HTA bodies agreeing to exchange their views, identify the key issues of the development proposed by the company, discuss their positions and try to reach consensus advice as far as possible. | SEED consortium  (collaborative approach from HTA bodies)  EMA, HTA agencies, companies, EuneHTA, European patients representatives and health professionals. | Drugs and medical devices. | The SEED consortium, gathering 14 HTA bodies, coordinated by HAS, has been selected and has been operated since May 2014 (first ED) to October 2015 (final report). | 11 EDs conducted (8 on drugs including 4 run in parallel with regulatory  scientific advice at the EMA and 3 on medical devices)  In addition to HTA bodies and companies, patients representatives and health professionals have been involved in some EDS. A short survey has been conducted after each ED, allowing participants to propose improvements to the procedure, and provide input for the permanent model | Early dialogue:  Single HTA advices  Parallel regulatory and HTA advices | Conference abstract  Considering the success of this activity (10 EUnetHTA EDs done instead of 3), the European Commission (DG SANTE) has published a call for tender for the conduct of 10 additional ‘multiple-HTA’ EDs and the production of a proposal for a permanent model for this activity in Europe.  . |
| Ng 2018  (Id 166) | To assess the impact of integrated SA (ISA) on clinical development and health technology assessment (HTA) of new medicines in Europe. | EMA and HTAbs | New medicines | Systematic review:  Publications (authored by EMA or HTAb representatives) that analysed the impact of ISA:   - Manufacturers compliance with the advice (impact on evidence generation programmes) - Outcome of Marketing Authorization (MA) - Reimbursement (THA appraisal) | 12 studies: six studies explored the impact of SA on evidence generation programmes, authored by EMA or HTAb representatives. The publications analysed previous SA meetings and explored questions asked by the manufacturers and level of compliance between stakeholders in a qualitative and quantitative manner.  There has been a marked increase in integrated scientific engagement in recent years. Manufacturers most frequently complied with EMA advice, followed by compliance with at least one HTAb.  SA was frequently followed for study design and least followed was comparator advice (57%) **where frequently the HTAb and EMA were not aligned**.  For parallel SA performed between 2010-2015, the products which gained MA, also gained positive reimbursement decisions from most of the HTAbs. For parallel and HTA-only SA, the majority of remaining products continued in clinical development and other products failed development. | Parallel Scientific Advice: to receive formal, consolidated feedback from the European Union (EU) regulators and HTAbs on their development plans for new medicines. | ISA offers a valuable dialogue opportunity for stakeholders from different perspectives. This analysis demonstrates that SA may increase manufacturers’ chance to obtain regulatory and reimbursement recommendation. Research from other stakeholders (e.g. patient representatives) will provide further understanding of the impact of SA. |
| Tafuri 2016 (Id 47) | To explore how the parallel scientific advice system is working and levels of  commonality between the EU regulators and HTABs, and among HTABs, when applicants obtain parallel scientific advice from both a regulatory and an HTA perspective. | Manufacturers, EMA and HTA bodies. | Medicines | Retrospective qualitative analysis.  Analysis of the minutes of discussion meetings held at the EMA between 2010, when parallel advice was launched, and 1 May  2015, when the cutoff date for data extraction was set. Analysis based on predefined criteria and conducted at two  different levels of comparison: answers of HTABs vs. regulators, and between participating  HTA agencies. | Analysis based on 31 procedures of parallel scientific advice. The level of full agreements was highest for questions on  patient population (77%); disagreements reached a peak for questions on the study comparator (30%). Regarding comparisons among HTABs, high level of agreement for all domains. | Parallel Scientific advice (PSA) | Conclusion:  Evident commonality, in terms of evidence requirements between the EU regulators and participating HTABs, as well as  among HTABs, on most aspects of clinical development. Indeed, regardless of the question content, the analysis showed that a high level of overall agreement was reached through the process of parallel scientific advice |
| Tafuri 2018  (Id 29)  (follow-up analysis of Tafuri 2016, id 47). | to investigate whether PSA is integrated in the clinical development programmes for which advice was sought. | Manufactures, EMA and HTA bodies | Medicines | -Extraction of the advice provided by the regulators and the HTA bodies for each procedure.  - Tracking the development of all the  clinical studies for which the PSA had been sought and in extracting information on the primary endpoint and comparator  used in such studies.  Three different sources were used: the EU Clinical Trial Register (https://www.clinicaltrialsregister.eu/), the National Institute of Health portal for clinical trials (https://clinicaltrials.gov/) and the AdisInsight database (http://adisinsight.springer.com). The cut-off date for data retrieval was 31 December 2016. Data were checked across the three databases to ensure accuracy and consistency | • Manufacturers tend to implement changes to the development programmes based on both regulatory and HTA advice with regards to the choice of primary endpoint and comparator.  • For the choice of the study comparator, manufacturers seem to be slightly more inclined to satisfy the regulatory  advice.  • Parallel scientific advice can greatly facilitate the integration of both regulatory and HTA perspectives into one clinical development, potentially reconciling their data requirements. | Parallel regulatory–health technology assessment scientific advice (PSA) | One of the key findings of this analysis is that manufacturers tend to implement changes to the development programme based on both regulatory and HTA advice with regards to the choice of primary endpoint and comparator. It also confirms the challenging choice of the study comparator, for which manufacturers seem to be more inclined to satisfy the regulatory advice. Continuous research efforts in this area are of paramount importance from a public health perspective |
| Vamvakas 2019  (Id 621) | To address clinical trials conducted for regulators and other decision-making purposes, the EMA offers different multistakeholder platforms (parallel advice with HTA bodies or with the FDA) in which different decision makers can provide simultaneous feedback on proposals for evidence generation.  We present the case for seeking such advice, how they work and which products are best targeted for which procedures. | EMA/HTAb | Pharmaceuticals. | Not defined. | 1. **Parallel advice between European Regulators and European HTA bodies:** Alignment between regulators and HTAs cannot be reached on the trial design in all cases given the different remits of respective organizations. The question then becomes “Can the remaining different evidence requirements be accommodated in a single trial?” A recent parallel procedure in gastroenterology exemplifies how this can be addressed. Although regulators would have been content with a placebo-controlled study, HTA bodies preferred two different active comparators. A consensus was reached that investigators’ best choice limited to three options would be acceptable to all parties. Other options for further discussion and resolution of divergences include follow-up procedures, qualification procedures for novel methodologies in drug development, or multistakeholder workshops at the EMA. In case of remaining divergences, knowLedge of the respective positions is essential for planning purposes. **Based on the advices received, the applicant will have to make design choices to address conflicting or diverging evidence requirements.** | Parallel advice (between European Regulators and HTAb)  Or Parallel Scientific Advice between EMA and FDA. | Only the information about the parallel advice between European Regulators and European HTA bodies is presented.  Most relevant products for parallel advice are those likely to be highly innovative, controversial, with wide public health implications, or a very restricted.  Since 2010:  -140 parallel advice procedures with HTA bodies have been undertaken showing consistent demand and a wide range of therapeutic areas.  - More than 50 parallel FDA advices undertaken.  It is too early to assess the impact of parallel advice at the time of MA or HTA appraisal given the lengthy development times or attrition of products in development but the EMA will continue to track these parallel procedures to further assess the impact. |
| Macaulay 2018  (Id 171) | This research reviews the available early dialogue services in major European markets, providing an overview of payer/HTA advice and highlights the remaining challenges for companies seeking to incorporate early HTA input | EuneHTA and EMA |  | Publicly-available data on early dialogue services provided by regulatory, payer and HTA bodies in Europe were reviewed, comparing the costs, duration, processes and outputs | The EMA and some European payer/HTA agencies have provided individual scientific advice since 2004. However, individual/national agency advice varies in duration (8-24+ weeks), processes (e.g., expertise used), price (~€2,000-€80,000) and outputs (e.g., report vs. meeting minutes). Stand-alone advice also requires separate company submissions. Parallel EMA/HTA advice was set up in 2011 to simplify the process of seeking pan-European advice requiring only a single document submission to all agencies. Despite the number of parallel advice projects steadily increasing since 2011, several areas of regulator and payer/HTA misalignment have been reported. The manufacturers were also left to consolidate individual agency reports. In a move to provide more coordinated advice, EUnetHTA Consolidated-Parallel-Consultation (EUnetHTA-CPC) builds on EMA/HTA parallel advice and provides a joint report of all agency positions, highlighting key differences. Despite joint advice output, the agencies nonetheless rely on different methodologies, often also reflecting variations in treatment pathways and/or comparator preferences. | Coordinated regulatory and payer/HTA advice.  Individual Scientific advice. | Authors from Parexel  Congress abstract.  Early advice options have improved significantly since 2011, with EUnetHTA-CPC procedure further supporting companies accessing the European market. However the challenges in reconciling differences in HTA methods and preferences remain. Furthermore, while EUnetHTA provides an overview of HTA opinions, it does not match the depth of advice provided by individual national advice, adding to the challenge of choosing the optimal early dialogue procedure(s). |
| Ofori-Asenso 2020 | To examine processes, progress, outcomes, and  challenges of harmonization/interaction initiatives between HTA bodies and regulatory  agencies. | EuneHTA, EMA, regulatory agencies and HTA bodies (see table 2) | Not specified (drugs and devices) | MEDLINE, EMBASE, and the International Pharmaceutical Abstracts database  were searched up to 21 October 2019. Searches for gray literature (working papers,  commissioned reports, policy documents, etc.) were performed via Google scholar  and several institutional websites. An online cross-sectional survey was also conducted  among HTA (n = 22) and regulatory agencies (n = 6) across Europe to supplement  the systematic review. | While there are areas of divergence,  there has been progress over time in narrowing the gap in evidentiary requirements for HTA bodies and regulatory agencies.  Regulatory agencies (4/6; 67%) and (11/22, 50%) HTA bodies reported having a formal link for “collaborating” with the other. Several mechanisms (early tripartite dialogues, parallel submissions  (reviews), adaptive licensing pathways, and postauthorization data generation have been explored as avenues for improving collaboration.  **In relation to early dialogues: tripartite early dialogues are described.**  Future approaches to improve harmonization/interaction between HTA bodies and regulatory agencies should build on these existing, examining their long-term impacts. Several barriers include legal, organizational, and resource-related factors. | Tripartite early dialogues: meetings among regulators, manufacturers and HTA bodies.  Joint (parallel) advice: advice given among regulatory agencies and HTA bodies related to unmet medical need, analysis methodology, acceptable primary  endpoints, etc.), discussing divergent data needs to minimize discrepancies and identifying trade-offs,  whereas manufacturers can have a single forum to discuss any  potential claims or concerns (52).  The opportunity to incorporate  patients’ and clinicians’ perspectives in these discussions could  further enrich the data needs (11, 53).  Scientific Advice from EMA; Early dialogue with HTA bodies  offers manufacturers: opportunity  to obtain early insight regarding evidence needs (e.g., safety,  efficacy/effectiveness, cost effectiveness, budgetary impact). | Hofer et al. revealed that from 2008 to 2012, 85% of  applications that received and followed EMAs’ early scientific advice were ultimately granted marketing authorization  compared to only 41% that did not (49).  Harmonizing this process via tripartite “early dialogue”  meetings consisting of regulators, manufacturers, and HTA  bodies can increase collaboration and improve understanding  among the different parties. Regulatory agencies and HTA bodies  can offer joint (parallel) advice (in areas such as defining |
| Schuurman S 2020  (Id 132) | to explore how US manufacturers could benefit from EU ISA. | EMA and HTA agencies | Pharmaceuticals (not stated, but if EMA takes part in the ISA…). | Review of internal case studies of ISA focusing on processes and results. The goal was to assess the advantages and potential barriers for US companies in engaging ISA based on our experience. | Alignment was achieved on EMA and HTA, and HTA-only questions on the CDP and economic analysis for a briefing book (BB). Key ISA areas of value were identified: de-risking the CDP and addressing uncertainty, managing internal expectations and eliminating the ‘siloing’ of teams by promoting early cross-functional collaboration and understanding of payer value drivers. Potential barriers: lack of HTA agencies able to provide advice within the set timelines, change in the treatment landscape between the time of seeking advice and the actual submission, and most agencies expecting to receive the BB before the start of Phase 2/3 studies. Finally, ISA is not binding and could be considered both as a barrier and an advantage to the manufacturer | Integrated Scientific Advice | Conference abstract  From the point of view of the industry, manufacturers would ideally adjust their clinical development plan strategy to the EU health technology assessment (HTA) requirements and obtain integrated scientific advice (ISA) to inform their CDPs.  Authors are members of ICON plc. |
| Galbraith M 2022 | To describe the process, results, and experiences of EUnetHTA Joint Action 3’s (JA3) Early Dialogue  (ED) activities and to highlight opportunities for improving the processes | EMA and HTA bodies (EunetHTA) | Pharmaceuticals | Descriptive analysis of the EUnetHTA ED process steps and evaluation of ED data conducted by EU HTA bodies, published  guidelines, and documents, as well as internal statistics. | Early Dialogues Working Party (EDWP) established in JA3.  From June 2017 to May 2021: 113 requests received, 38 conducted (32 Parallel consultations and 6 multi-HTA dialogues).  Exchanges between EMA and HTAbs at multiple stages prior to the meeting with the applicant (added value compared to previous parallel advice).  • Modifications of development plans were proposed by applicants in twelve out of twenty-one (57 percent) EDs after receiving the EUnetHTA (in design adaptation, primary end point choice, changes  of comparator, and the addition of a study, but also population criteria, intervention, and other outcomes.  Population, intervention, comparator, and outcome (PICO) framework provides a standard format for specifying research questions.  Health economics addressed in more than half, and post-launch evidence generation (PLEG) in one third.  High percentage of alignment between HTAbs (>80% full alignment on all PICO items)  Process continuously optimized.  Different approaches tested for patient involvement. 85% EDs in JA3 involved patients  Finally, a centralized procedure was chosen with the key documents produced by two responsible agencies and reviewed by the EDWP.  Patient involvement primarily done by interviewing a national patient representative to obtain general feedback on the disease and the planned study design.  Most big pharma companies, only 15% SME. | Parallel consultations  Mutli-HTA Early Dialogue. | From 4 to 9 HTAbs taking part in each ED procedure (average 6.7).  Projects and Joint Actions function on limited and strict budgets. Although an Early Dialogues Financing  Mechanism (EDFM) was developed and a framework for a fee-for service model, it could not be piloted during JA3. |

**Supplemental Material 4: Scientific Advice/Early dialogue services by organization**

**Table 1**: Scientific advice/early dialogue services by individual HTA bodies

| **Organization/s** | **References** | **Type of process (EA/SA/ED)**  **Definition** | **Type of technology** | **Process definition (phases, length of the meetings, language and time needed)** | **Involved stakeholders (who and how participates)** | **Fees** | **Output** | **Impact (improvement, acceptance and added value/satisfaction with the final product)** |
| --- | --- | --- | --- | --- | --- | --- | --- | --- |
| NICE (UK) | (4, 7, 14, 16, 17, 19, 21, 24, 26, 27)  **Website:**  <https://www.nice.org.uk/about/what-we-do/life-sciences/scientific-advice> | Different options of scientific advice:  • Standard scientific advice  • Express scientific advice  Parallel services with regulatory agencies or other HTA bodies also available. | Pharmaceuticals and Medtech (devices, diagnostics, digital technologies). | **1. Devices, diagnostics, and digital technologies:**  *1.1. META Tool*  Online tool to identify gaps in the evidence base and steps to bring company’s product to market.  *1.2. Medtech advice*  Combining META Tool with scientific advice service to:  • identify potential gaps in evidence base  • advise on key issues for evidence generation plans.  *1.3. Standard scientific advice*  Detailed clinical and economic advice to ensure sufficient evidence generation plans for the technology.  Input from a panel of experts  **2. Pharmaceutical products**  Number of services for timing /budget flexibility:  *2.1. NICE advice services*  Standard scientific advice: detailed clinical and economic advice from NICE and a panel of experts (18 weeks)  Express scientific advice: accelerated version of standard scientific advice service for immediate need (12 weeks).  *2.1. Parallel advice services* (with CADTH or MHRA, or European Concurrent advice with EMA).  **3. Preliminary Independent Model Advice (PRIMA)**  Expert independent advice to improve economics models.  (peer review service). | Depending on the needs:  • a clinician  • a health economist  • an HTA expert  • a patient expert or carer.  Regulators also if required. | Standard advice: £38,024- 91,051  Express advice: £49,431-82,632  Concurrent advice: £31,221-84,397  Fixed fees for MedTech Advice (£15,000) and META tool (£3,500).  Small/medium enterprises can have more affordable options, starting from £ 29,000. | Report that helps to:  1. Develop evidence that shows the value of the product  2. Detailed feedback on evidence generation plans  3. Helps to build cost-effectiveness measures into evidence generation plans. | 4 articles assessing type of SA and impact (7, 16, 26, 27).  SA between 2009 and 2015: most frequently addressed questions related to clinical development and main pivotal efficacy studies.  Parallel advice procedures focus on clinical efficacy issues; cost-effectiveness tend to dominate in NICE only procedures.  Issues mostly discussed in the HTA SA: comparator, generalisability of trial evidence to NHS practice and impact of trial outcomes on quality of life and survival.  Less disagreement with clinical endpoints, population definition, position of the technology in treatment pathway and study design (7). |
| HTW (Wales) | (13)  **Website:** <https://healthtechnology.wales/sas/#:~:text=The%20HTW%20Scientific%20Advice%20Service,providers%2C%20patients%20and%20service%20users>. | HTW Scientific Advice Service (expert consultancy to support Wales developers/ innovators to generate evidence and demonstrate value that meets the needs of care commissioners, providers, patients and service users). | Non-medicine health technologies. | The HTW Scientific Advice Service is an expert consultancy that supports developers and innovators in Wales to generate evidence and demonstrate value that meets the needs of care commissioners, care providers, patients and service users. 5 days to decide if is in HTWs’ scope, and 10 days  SAS complete procedure: 6-8 weeks | Not reported. | No data. | Identify gaps in evidence, support evidence generation activities, save time and resource, and help a product to market. | No data available. |
| CADTH (Canada) | (14, 15)  **Website:**  <https://www.cadth.ca/frequently-asked-questions-0> | [Scientific Advice](https://www.cadth.ca/scientific-advice) Advice to pharmaceutical companies on early drug development plans from an HTA perspective.  Early Parallel Scientific Advice most beneficial for drugs for rare diseases/conditions and other challenging clinical populations; new therapeutic areas; complex, adaptive or unusual trial designs; or development plans that may include use of real-world evidence. | Pharmaceuticals (new drug products,  existing drug products with new indications,  drugs for rare diseases,  oncology products). | CADTH SA Program: voluntary, non-binding, confidential fee-for-service consultation.  **1. Submit Application**1.1 Submit online application 4 weeks prior to Briefing Book submission. 1.2 Application assessed for eligibility. 1.3 If eligible, confirmation of acceptance and the first invoice send to applicant.  **2. Submit Briefing Book (BB)** 2.1 Submission 14 weeks prior to SA Meeting 2.2 Applicant receives the BB clarification questions. 2.3 Applicant receives second invoice 6 weeks prior to SA Meeting.  **3. Attend Scientific Advice Meeting** Three hours face-to-face meeting. CADTH discusses the advice with the applicant based on the questions submitted in the BB.  **4. Receive Record of Scientific Advice** CADTH Record of Scientific Advice (ROSA) 4.1 Written ROSA received for the applicant 4 weeks after SA Meeting. 4.2 Applicant submits ROSA clarification questions (optional). 4.3 Applicant receives final invoice. | Not reported. | From 65,000 to 100,000 CAN $.  (Subjected to applicable taxes).  Total fee determined after submission of Briefing Book. Updated fee schedule provided within one week of the briefing book submission date. | ROSA (Record of Scientific advice). | No data available. |
| G-BA (Germany)/IQWIC | (4, 19, 21, 23, 24, 28) (innovative medical devices: IQWIC)  (17)  (parallel advice con BfArM) | HTA Scientific advice, within the early benefit assessment (EBA) of pharmaceuticals | New drugs  Innovative Medical devices (IQWIC) | FJC commissions IQWiG to prepare evidence reports on pharmaceuticals and non-drug interventions and assesses EBA dossiers of new pharmaceuticals.  A pre-structured matrix to requests company’s contact details, active substance description, product’s market authorization status, list of annexes, and questions for discussion.  National approval authorities (BfArM) and Paul Ehrlich Institute (PEI)) can be involved in the SA process by written request from manufacturer.  Scientific advice can be “early” (manufacturers can modify product development) or “late,” (to offer precise information on EBA requirements).  Table showing timeline for consultation request.  Pre-register for selected consultation appointments providing information on the active ingredient itself and planned indication (including details such as therapy line) (in german in: https://www.g-ba.de/themen/arzneimittel/arzneimittel-richtlinie-anlagen/nutzenbewertung-35a/informationen-fuer-unternehmen/#Beratungsslots).  German form for consultation request.  For consultations dealing with study design, additional form, either in German or English.  2 months time (4) | Involved units of FJC, administrative office of FJC, responsible subcommittee for pharmaceuticals, specific working group for EBA and specialist counselling group Medicine with respective methodical expertise. | From €2,000 for general enquires on the rules to €7,000 for necessary trial data to be submitted within the EBA.  €10,000 for appropriate comparative therapy (ACT).    Advance payment of 5,000 € required to initiate SA.  (fee schedule from 9 December 2021 (available in German at: https://www.g-ba.de/beschluesse/5116/ ). | Meeting minutes (4) | Dintsios (28) described manufacturers experience with the SA offered by FJC.  Manufacturers criticized inconsistencies in the process, lacking expertise with conducting clinical trials, partially incomplete answers and a low readiness to engage in dialogue.  Majority of responders show positive attitude concerning unambiguousness, completeness, traceability, atmosphere and protocol of advice.  Positive trend in industry’s perception of the SA over time.  More active involvement of additional stakeholders and the incorporation of procedural elements from other healthcare systems could improve the quality of the SA offered by the FJC.  Plaud et al (23) indicated that IQWG has an early dialogue framework to ensure patients fast access to safe and effective medical innovation. |
| HAS (France) | (12, 19, 21, 24)  **Website:** <https://www.has-sante.fr/jcms/c_2623726/en/guidance-for-national-early-dialogues-on-medicinal-products> | Early dialogue (ED)  Questions for ED:  a. Population included and generalisability with res­pect to the claimed indication  b. Clinical trial comparator and/or other relevant comparator(s)  c. primary/ secondary endpoints (including PROs)  d. Design and/or statistical analysis  e. Data collection after MA granted (optional)  f. Medico-economic assessment (optional)  g. Other questions (optional) | Innovative Medicinal products | ED with companies developing innovative medicinal products or other technologies that have a new mechanism of action, are targeting an insufficiently covered medical need and if the request is submitted before the start of pivotal clinical trials.  ED optional, confidential, and not binding for either HAS or pharmaceutical companies.  Discussions and documents can be submitted in English.  HAS staff obliged to maintain professional secrecy (also other stakeholders involved).  HAS staff participating in ED have no conflicts of interest with the technology assessed.  Experts participating not able to participate in future assessment and appraisal.  ED as standard (face-to-face meeting) or accelerated procedure (without face-to-face meeting). Timeline depends on submission date, in accordance with an ED calendar published on HAS website.  For standard procedure: -37 days for request; +7 days to determine choice of procedure; +45 days list of issues from HAS;+90 face to face meeting; +100 minutes sent; +110 final recommendations.  For accelerated procedure: -37 days for request; +7 days to determine choice of procedure; +60 days for written recommendations; +70 for clarifications; +75 days finished. | HAS staff  Experts and patients who may be involved at the request of HAS. | No fees. | Recommendations on last development phase and support generation of good quality evidence for proper HTA quality assessment. | (12)  84 early dialogues performed by HAS (53 with the EMA and/or others European HTA bodies),  Mainly focused on phase III trials.  Following ED, the clinical study not yet implemented in 25 cases.  When clinical trial was effectively launched, 10 cases with – results, + in 11 and ongoing for 29.  Only eight medicinal products appraised by HAS (all with clinical added value score).  Success rate of clinical development for products that underwent an early dialogue higher than data from literature, although it was likely to decrease in follow-up analysis. |
| ZIN (Before 2014 named as CVZ) | (4)  **Website:** <https://english.cbg-meb.nl/topics/mah-scientific-and-regulatory-advice> | Scientific Advice (SA) In parallel with regulatory agency MEB.  **Different options:**   - Simple advice (regulatory advice, pharmaceutical or pre-clinical aspects or follow up advice) - Partial multi-disciplinary SA: clinical advice (safety/ efficacy) only or with pharmaceutical or preclinical advice… - Complete multi-disciplinary SA: (clinical, preclinical and pharmaceutical advice) - Customised advice (small companies: pharmaceutical/ preclinical aspects of a medicinal product in early development phase). | Medicinal products | MEB provides scientific and regulatory advice and enables to improving the medicine-development process so that it is as efficient and responsible as possible.  <https://english.zorginstituutnederland.nl/latest/news/2019/05/09/cbg-meb-and-zin-have-started-the-%E2%80%9Ccbg-zin-parallel-procedures-pilot%E2%80%9D-to-accelerate-access-to-innovative-medicines>   - Application form validated and companies notified within three weeks as to whether the application can be accepted. - Written advice within 7 weeks from full documentation receipt - Oral advice: meeting between 6 weeks and 3 months after accepting application: documentation, presentation and list of attendess sent three weeks before the meeting - Meeting will last 90 min. - Company writes a report on the meeting and sends it to MEB 2 weeks following the meeting - MEB sends final advice within 4 weeks of the meeting. - If applicant still has questions after receiving the advice, further clarification could be requested but the advice will not be reviewed again. | MEB staff.  Clinicians and staff from ZIN could participate (if selected by the company in the application form). | **Scientific advice:**   - Simple advice 6,470 € (written answer). - Partial multi-disciplinary SA 11,070 €. - Complete multi-disciplinary advice: 14,760 €. - Customised advice: 2,180 euros. | Written advice. | No data available. |
| TLV (Dental and Pharmaceuticals Benefit Agency) | (4, 17, 21)  **Website**:  <https://www.lakemedelsverket.se/en/permission-approval-and-control/advisory/scientific-advice#hmainbody4> | Scientific advice (SA).  SA may concern the following areas: non-clinical, clinical, statistics, Pharmacokinetics, Quality/Chemistry, Quality/Biotechnology or Regulatory/scientific questions | Pharmaceuticals | SA from TLV can be asked when a company is seeking SA by the Swedish regulatory agency (MPA).  Applicant may request any form of SA, but the Swedish MPA determines the form depending on the nature of the questions. The length of the meeting is 90 minutes.  The applicant may submit their meeting minutes within a month to the Swedish MPA. The Swedish MPA will acknowledge receipt of the minutes and may comment on the content. The minutes cannot be interpreted as a document issued by the Swedish MPA, or as an official position of the Agency. | Company can select in the application form the expertise needed, including participants from the TLV. | No additional fee to that of the MPA SA.  SA by MPA is about 6239,6 €. | Minutes | No data available. |
| AIFA | (10, 19, 21)  **Website:**  <https://www.aifa.gov.it/documents/20142/648668/AIFA_guidance_applicants_requesting_Scientific-Advice_28.11.2017.pdf>  <https://www.aifa.gov.it/en/innovazione-e-scientific-advice>). | Scientific advice  Issues related to:  1. Most appropriate comparator  2. Endpoings  3. Population  Generally, products in Phase II (likely to be authorized after Phase II) and Phase III.  Innovative meetings | Pharmaceuticals. | Services suspended on 21 December 2021 (see website).  Scientific advice  Briefing book required (with list of questions and company position for each one).  HTA aspects related to efficacy and relative effectiveness evaluation.  Applicants should fill in a predefined form (with rationale, questions etc).  Within 20 working days, SA Coordinator notifies by email if request is acceptable  After 5 working days, AIFA Evaluation Team defined and contract drafted including fee due to AIFA  Report in 90 days from the contract signing.  A preliminary version discussed after 60 days, within a meeting or a teleconference.  They generally refer to products in Phase II (likely to be authorized after Phase II) and Phase III.  Company sends the minutes of the meeting. AIFA sends final written report.  Innovative meetings  Informal meetings to present an innovative product, technology or methodology to receive feedback or guidance on the evolution of the development programme (available at: <https://www.aifa.gov.it/en/innovazione-e-scientific-advice>). Could be requested by pharmaceutical industries, small and medium-sized enterprises, Universities and Academic Institutions and Research Institutions. | AIFA staff | For Scientif advice:  Fee from €10k to €40k (4). | Final written report for scientific advice | Current uptake of this service by manufacturers in Italy is not publicly available (10). |

**Table 2**: Early dialogue/joint advice services by EunetHTA and EMA

| **Organization/s** | **References** | **Type of process (EA/SA/ED)**  **Definition** | **Type of technology** | **Process definition (phases, length of the meetings, language and time needed)** | **Involved stakeholders (who and how participates)** | **Fees** | **Output** | **Impact (improvement, acceptance and added value/satisfaction with the final product)** |
| --- | --- | --- | --- | --- | --- | --- | --- | --- |
| EMA-EuneHTA | (4, 8, 11, 17, 18, 20, 25, 29-31) | ED  ED: non-binding SA, before the start of pivotal clinical trials (after feasibility/ proof of concept study), to improve the quality and appropriateness of the data produced by the developers in view of future HTA assessment / re-assessment. | Mainly drugs (although multi-HTA ED was offered to devices at the beginning). | Two types:  Parallel consultation (current Joint Scientific Consultations): EMA and HTA bodies (only for pharmaceuticals)  Multi-HTA:  For pharmaceuticals and devices  EUnetHTA selection criteria (bring added benefit to patients, i.e. by:   - A new mode of action for the indication; - *AND* debilitating disease; - *AND* responding to unmet need (no treatment or only unsatisfactory treatment available). - targeting a life-threatening or chronically.   **Phases of ED:**  Prenotification phase: applicant sends a letter of intent (formal template). 5-10 days to communicate the decision of EUnetHTA to the applicant.  Drafting the briefing book:   - Applicant sends a draft briefing book 30 days after sending the letter. - Request for clarification by EUnetHTA members 15 days after. - Final draft confirmed by EUnetHTA in 15 days.   Evaluation phase:  EUNEHTA sends list of issues for clarification 30 days after receiving the briefing book, with 15 days for answering them.  Applicant has 15 days to answer the list of issues.  Face to face meeting and finalisation:  Meeting 60 days after sending the briefing book.  10 days to write the final recommendations for HTA bodies  5 days after (75 days from the sending of the briefing book) EUnetHTA sends final consolidated HTA-ED written answers to applicant. | Depending on the asked service:   - Different HTA bodies interested in the consultation. - EMA   Hearing directly from patients is important from an HTA perspective. The procedures for how external stakeholders are engaged are available from the EUnetHTA ED Secretariat. | No clear. There are fees, but not specified. | Report offering the recommendations in an individual manner | HTA bodies with highest representation in 43 analysed parallel procedures: NICE (90%), G-BA (65%), AIFA (45%), TLV (Sweden) (35%), HAS (19%) (9).  Analysis of questions and answers by regulators and HTA assessors in 31 parallel procedures among 2010-2015: full agreements reached in 61%, partial agreements in 23%, disagreements in 16% (9)  Divergence higher regarding comparator (9).  Manufacturer implemented comparators recommendations to address both the needs of regulators and at least one HTA body in 12 out of 21 studies (60%) (9). Manufacturers following the regulators’ and >50% HTA bodies advice: 8/21 (38%).  Those following exclusively regulatory advice: 7/21.  Changes never implemented solely based on the HTA advice.  For primary endpoint in all included studies (n=23), manufacturers implemented both the requests of the regulators and at least one HTA body.  In 15 studies the manufacturer complied with the advice of both the regulators and more than 50% of the HTA bodies. |
